# Supplementary material for: From eggs to bites: do ovitrap data provide reliable estimates of Aedes albopictus biting females?
Source: PeerJ. 2017 Mar 16;5:e2998. doi: 10.7717/peerj.2998 (PMC5357344; doi:10.7717/peerj.2998)
Supplement: Table S1 — Models were ranked by AICc using maximum likelihood estimation (ML). [file peerj-05-2998-s008.pdf]

Table S1. Results of Multi-model selection approach (Model-I). Models were ranked by AICc using maximum likelihood estimation (ML)

Intercept	Mean number/ eggs/day	Mean number/eggs/day lag 1	Mean number/eggs/day lag 2	Rain	Site	Temperature	Temperature^2	Wind	df	logLik	AICc	delta	weight
14.71	0.24							-1.88	5.00	-237.35	485.62	0.00	0.03
14.77	0.23								4.00	-238.54	485.69	0.07	0.03
12.14	0.17	0.22						-2.01	6.00	-236.37	486.06	0.44	0.02
17.08	0.22					0.11	-0.16		6.00	-236.42	486.16	0.54	0.02
17.03	0.24					-0.39	-0.20	-2.24	7.00	-235.21	486.19	0.57	0.02
14.84	0.24			+					5.00	-237.70	486.32	0.70	0.02
15.34	0.21					0.54			5.00	-237.71	486.35	0.73	0.02
12.33	0.17	0.21							5.00	-237.72	486.37	0.75	0.02
11.99	0.23		0.17						5.00	-237.85	486.63	1.01	0.02
12.17	0.18	0.23		+					6.00	-236.67	486.66	1.04	0.02
17.27	0.24			+		-0.17	-0.19		7.00	-235.46	486.70	1.08	0.02
12.17	0.23		0.16					-1.82	6.00	-236.75	486.81	1.19	0.02
12.84	0.15	0.21				0.56			6.00	-236.82	486.95	1.33	0.01
16.60	0.24				+			-1.90	6.00	-236.89	487.09	1.47	0.01
13.99	0.17	0.25			+			-2.05	7.00	-235.67	487.12	1.50	0.01
16.67	0.24				+				5.00	-238.11	487.14	1.52	0.01
14.77	0.24			+				-1.56	6.00	-236.92	487.15	1.53	0.01
18.93	0.25				+	-0.54	-0.22	-2.46	8.00	-234.45	487.22	1.60	0.01
17.23	0.26			+		-0.57	-0.22	-2.03	8.00	-234.46	487.24	1.62	0.01
12.06	0.18	0.23		+				-1.65	7.00	-235.81	487.40	1.78	0.01
12.19	0.24		0.16	+					6.00	-237.06	487.43	1.81	0.01
14.17	0.17	0.23			+				6.00	-237.07	487.45	1.83	0.01
12.73		0.37				0.70			5.00	-238.27	487.46	1.84	0.01
13.71	0.23		0.21		+				6.00	-237.10	487.50	1.88	0.01
18.98	0.23				+	0.02	-0.17		7.00	-235.86	487.51	1.89	0.01

11.89		0.41						-2.07	5.00	-238.31	487.55	1.93	0.01
10.19	0.17	0.20	0.14					-1.92	7.00	-235.89	487.55	1.93	0.01
10.10	0.17	0.19	0.15						6.00	-237.12	487.56	1.94	0.01
15.04	0.22					0.31		-1.46	6.00	-237.12	487.56	1.94	0.01
14.05	0.18	0.25		+	+				7.00	-235.98	487.74	2.12	0.01
15.26	0.22			+		0.42			6.00	-237.21	487.74	2.12	0.01
13.86	0.24		0.19		+			-1.84	7.00	-235.98	487.74	2.12	0.01
14.82	0.21		0.13			0.04	-0.15		7.00	-235.99	487.75	2.13	0.01
15.05	0.17	0.15				0.18	-0.13		7.00	-236.00	487.78	2.16	0.01
12.93	0.21		0.14			0.46			6.00	-237.24	487.80	2.18	0.01
16.75	0.25			+	+				6.00	-237.25	487.81	2.19	0.01
14.88	0.23		0.12			-0.42	-0.19	-2.22	8.00	-234.76	487.84	2.22	0.01
19.22	0.25			+	+	-0.30	-0.20		8.00	-234.77	487.86	2.24	0.01
15.15	0.19	0.14				-0.32	-0.17	-2.24	8.00	-234.78	487.89	2.27	0.01
12.06		0.40							4.00	-239.65	487.90	2.28	0.01
17.22	0.22				+	0.52			6.00	-237.32	487.94	2.32	0.01
12.46	0.16	0.22				0.31		-1.58	7.00	-236.14	488.06	2.44	0.01
10.11	0.18	0.21	0.14	+					7.00	-236.14	488.06	2.44	0.01
19.18	0.27			+	+	-0.75	-0.24	-2.25	9.00	-233.57	488.10	2.48	0.01
12.57	0.16	0.23		+		0.43			7.00	-236.16	488.10	2.48	0.01
11.85	0.17	0.21	0.18		+				7.00	-236.18	488.13	2.51	0.01
11.94	0.17	0.22	0.17		+			-1.95	8.00	-234.92	488.16	2.54	0.01
15.19	0.19	0.16		+		-0.10	-0.16		8.00	-234.96	488.25	2.63	0.01
14.71	0.15	0.23			+	0.53			7.00	-236.24	488.27	2.65	0.01
16.51	0.25		0.16		+	-0.60	-0.20	-2.44	9.00	-233.68	488.31	2.69	0.01
15.07	0.24		0.13	+		-0.22	-0.18		8.00	-235.01	488.34	2.72	0.01
13.82		0.43			+			-2.13	6.00	-237.52	488.36	2.74	0.01
13.90	0.25		0.20	+	+				7.00	-236.29	488.36	2.74	0.01
11.92		0.42		+					5.00	-238.76	488.45	2.83	0.01
12.29	0.24		0.15	+				-1.51	7.00	-236.34	488.45	2.83	0.01
13.94	0.17	0.26		+	+			-1.68	8.00	-235.09	488.49	2.87	0.01

16.42	0.22		0.17		+	-0.09	-0.16		8.00	-235.13	488.59	2.97	0.01
17.00	0.19	0.17			+	-0.50	-0.19	-2.51	9.00	-233.84	488.62	3.00	0.01
10.90	0.15	0.20	0.13			0.49			7.00	-236.43	488.63	3.02	0.01
14.67		0.39			+	0.66			6.00	-237.66	488.64	3.02	0.01
11.89	0.17	0.23	0.17	+	+				8.00	-235.17	488.66	3.04	0.01
16.67	0.25			+	+			-1.58	7.00	-236.45	488.68	3.06	0.01
13.97		0.42			+				5.00	-238.91	488.75	3.13	0.01
12.40		0.38				0.48		-1.40	6.00	-237.76	488.84	3.22	0.01
15.30	0.20	0.15		+		-0.50	-0.19	-2.01	9.00	-233.97	488.89	3.27	0.01
9.91		0.38	0.14					-1.97	6.00	-237.79	488.90	3.28	0.01
12.51		0.39		+		0.59			6.00	-237.80	488.91	3.29	0.01
14.44		0.33				0.42	-0.10		6.00	-237.80	488.92	3.30	0.01
16.89	0.17	0.17			+	0.08	-0.14		8.00	-235.30	488.93	3.31	0.01
16.73	0.25		0.16	+	+	-0.39	-0.20		9.00	-233.99	488.94	3.32	0.01
9.79		0.37	0.16						5.00	-239.01	488.94	3.32	0.01
14.55	0.22		0.18		+	0.41			7.00	-236.59	488.96	3.34	0.01
15.16	0.25		0.12	+		-0.59	-0.21	-1.99	9.00	-234.02	488.99	3.37	0.01
12.60	0.22		0.15			0.23		-1.51	7.00	-236.62	489.02	3.40	0.01
10.18	0.17	0.22	0.13	+				-1.58	8.00	-235.35	489.03	3.41	0.01
11.82		0.42		+				-1.74	6.00	-237.87	489.05	3.43	0.01
10.80		0.35	0.13			0.63			6.00	-237.88	489.07	3.45	0.01
17.11	0.19	0.18		+	+	-0.25	-0.18		9.00	-234.09	489.13	3.51	0.00
16.91	0.23				+	0.29		-1.51	7.00	-236.69	489.16	3.54	0.00
12.84	0.22		0.14	+		0.35			7.00	-236.73	489.23	3.61	0.00
11.78		0.39	0.17		+			-2.00	7.00	-236.75	489.27	3.65	0.00
11.66		0.38	0.19		+				6.00	-237.99	489.29	3.67	0.00
13.88		0.44		+	+				6.00	-237.99	489.29	3.67	0.00
14.29	0.16	0.24			+	0.27		-1.67	8.00	-235.49	489.30	3.68	0.00
16.85	0.27		0.15	+	+	-0.76	-0.23	-2.19	10.00	-232.82	489.30	3.68	0.00
15.02	0.23			+		0.24		-1.27	7.00	-236.78	489.34	3.72	0.00
17.15	0.23			+	+	0.40			7.00	-236.79	489.36	3.75	0.00

14.46	0.16	0.25		+	+	0.39			8.00	-235.53	489.38	3.76	0.00
17.24	0.21	0.17		+	+	-0.69	-0.22	-2.26	10.00	-232.87	489.42	3.80	0.00
13.97	0.24		0.19	+	+			-1.52	8.00	-235.57	489.46	3.84	0.00
12.58	0.15	0.22	0.16		+	0.43			8.00	-235.62	489.55	3.93	0.00
13.24	0.17	0.13	0.12			0.12	-0.13		8.00	-235.64	489.60	3.98	0.00
12.30	0.16	0.23		+		0.24		-1.35	8.00	-235.67	489.67	4.05	0.00
11.96	0.17	0.23	0.16	+	+			-1.59	9.00	-234.37	489.69	4.07	0.00
9.81		0.39	0.15	+					6.00	-238.19	489.69	4.07	0.00
14.54		0.35				0.00	-0.13	-1.90	7.00	-236.97	489.72	4.10	0.00
10.57	0.16	0.21	0.13			0.25		-1.59	8.00	-235.74	489.79	4.18	0.00
13.52	0.19	0.12	0.11			-0.34	-0.16	-2.19	9.00	-234.44	489.83	4.21	0.00
10.69	0.16	0.22	0.12	+		0.37			8.00	-235.77	489.86	4.24	0.00
12.58		0.37	0.16		+	0.57			7.00	-237.04	489.86	4.24	0.00
13.78		0.44		+	+			-1.79	7.00	-237.06	489.90	4.28	0.00
14.31		0.40			+	0.42		-1.51	7.00	-237.08	489.93	4.31	0.00
16.40		0.35			+	0.31	-0.11		7.00	-237.10	489.98	4.36	0.00
14.54		0.36		+		0.21	-0.12		7.00	-237.12	490.03	4.41	0.00
14.46		0.41		+	+	0.54			7.00	-237.14	490.05	4.43	0.00
11.71		0.41	0.18	+	+				7.00	-237.14	490.06	4.44	0.00
14.19	0.23		0.18		+	0.18		-1.59	8.00	-235.90	490.13	4.51	0.00
15.22	0.20	0.14	0.14		+	-0.52	-0.18	-2.43	10.00	-233.25	490.17	4.55	0.00
13.51	0.19	0.14	0.11	+		-0.14	-0.15		9.00	-234.61	490.18	4.56	0.00
14.89	0.17	0.15	0.15		+	-0.01	-0.13		9.00	-234.69	490.34	4.72	0.00
14.46	0.23		0.18	+	+	0.30			8.00	-236.04	490.39	4.77	0.00
16.55		0.37			+	-0.18	-0.15	-2.18	8.00	-236.05	490.43	4.81	0.00
10.54		0.37	0.13			0.42		-1.40	7.00	-237.37	490.51	4.89	0.00
9.91		0.40	0.14	+				-1.66	7.00	-237.38	490.53	4.91	0.00
10.63		0.37	0.13	+		0.53			7.00	-237.41	490.60	4.98	0.00
12.23	0.16	0.22	0.16		+	0.18		-1.69	9.00	-234.83	490.61	4.99	0.00
15.26	0.19	0.15	0.14	+	+	-0.30	-0.17		10.00	-233.49	490.65	5.03	0.00
12.27		0.40		+		0.42		-1.20	7.00	-237.44	490.65	5.03	0.00

12.60		0.32	0.12			0.37	-0.09		7.00	-237.45	490.67	5.05	0.00
12.39	0.16	0.23	0.16	+	+	0.31			9.00	-234.89	490.73	5.11	0.00
12.58	0.23		0.15	+		0.17		-1.31	8.00	-236.27	490.86	5.24	0.00
16.59		0.38		+	+	0.06	-0.14		8.00	-236.28	490.89	5.27	0.00
14.16	0.16	0.26		+	+	0.20		-1.43	9.00	-234.98	490.92	5.30	0.00
11.80		0.41	0.17	+	+			-1.68	8.00	-236.31	490.95	5.33	0.00
13.79	0.20	0.13	0.10	+		-0.50	-0.19	-1.96	10.00	-233.66	490.98	5.36	0.00
16.90	0.24			+	+	0.22		-1.31	8.00	-236.33	490.99	5.37	0.00
15.58	0.21	0.14	0.13	+	+	-0.68	-0.20	-2.16	11.00	-232.34	491.15	5.53	0.00
12.30		0.38	0.16		+	0.34		-1.52	8.00	-236.44	491.21	5.59	0.00
14.66		0.36		+		-0.13	-0.15	-1.70	8.00	-236.46	491.24	5.62	0.00
12.43		0.39	0.16	+	+	0.46			8.00	-236.51	491.35	5.73	0.00
14.41		0.33	0.15		+	0.25	-0.10		8.00	-236.52	491.36	5.74	0.00
10.46	0.16	0.22	0.12	+		0.18		-1.35	9.00	-235.27	491.49	5.87	0.00
12.87		0.33	0.11			-0.01	-0.12	-1.84	8.00	-236.64	491.61	5.99	0.00
14.20		0.42		+	+	0.37		-1.30	8.00	-236.71	491.75	6.13	0.00
16.75		0.39		+	+	-0.34	-0.17	-1.98	9.00	-235.42	491.79	6.17	0.00
12.81		0.34	0.11	+		0.18	-0.11		8.00	-236.79	491.89	6.28	0.00
14.75		0.34	0.14		+	-0.19	-0.14	-2.08	9.00	-235.52	491.98	6.37	0.00
14.18	0.24		0.18	+	+	0.12		-1.38	9.00	-235.53	492.01	6.39	0.00
18.75						0.80			4.00	-241.75	492.11	6.49	0.00
20.64						0.45	-0.15		5.00	-240.66	492.24	6.62	0.00
12.15	0.16	0.23	0.16	+	+	0.12		-1.44	10.00	-234.33	492.32	6.70	0.00
14.71		0.35	0.14	+	+	0.02	-0.13		9.00	-235.73	492.40	6.79	0.00
10.44		0.38	0.12	+		0.37		-1.19	8.00	-237.04	492.41	6.79	0.00
12.23		0.40	0.15	+	+	0.30		-1.30	9.00	-236.08	493.12	7.50	0.00
18.37									3.00	-243.43	493.22	7.60	0.00
13.07		0.34	0.10	+		-0.13	-0.14	-1.64	9.00	-236.15	493.26	7.64	0.00
16.05			0.16			0.72			5.00	-241.25	493.42	7.80	0.00
15.05		0.36	0.13	+	+	-0.32	-0.16	-1.86	10.00	-234.93	493.52	7.90	0.00
17.96			0.16			0.35	-0.15		6.00	-240.15	493.61	7.99	0.00

18.36								-1.73	4.00	-242.51	493.62	8.00	0.00
20.81						0.17	-0.17	-1.40	6.00	-240.19	493.69	8.07	0.00
15.00			0.21						4.00	-242.62	493.84	8.22	0.00
20.55					+	0.80			5.00	-241.51	493.95	8.33	0.00
22.46					+	0.42	-0.16		6.00	-240.35	494.02	8.40	0.00
18.68						0.67		-0.87	5.00	-241.56	494.03	8.41	0.00
20.91				+		0.33	-0.17		6.00	-240.40	494.11	8.49	0.00
18.81				+		0.75			5.00	-241.65	494.23	8.61	0.00
15.19			0.20					-1.68	5.00	-241.76	494.45	8.83	0.00
18.52				+					4.00	-243.05	494.70	9.08	0.00
17.74			0.19		+	0.70			6.00	-240.82	494.94	9.32	0.00
20.17					+				4.00	-243.20	495.00	9.38	0.00
19.62			0.19		+	0.29	-0.15		7.00	-239.62	495.01	9.39	0.00
18.08			0.16			0.07	-0.17	-1.47	7.00	-239.63	495.04	9.43	0.00
16.77			0.24		+				5.00	-242.12	495.16	9.54	0.00
15.94			0.17			0.58		-0.93	6.00	-241.03	495.37	9.75	0.00
20.15					+			-1.74	5.00	-242.26	495.45	9.83	0.00
15.23			0.20	+					5.00	-242.27	495.46	9.84	0.00
22.64					+	0.13	-0.18	-1.46	7.00	-239.85	495.48	9.86	0.00
18.21			0.16	+		0.23	-0.16		7.00	-239.87	495.51	9.89	0.00
16.10			0.16	+		0.67			6.00	-241.14	495.60	9.98	0.00
18.45				+				-1.54	5.00	-242.37	495.66	10.04	0.00
21.03				+		0.10	-0.18	-1.27	7.00	-240.02	495.82	10.20	0.00
16.92			0.23		+			-1.69	6.00	-241.26	495.84	10.22	0.00
20.47					+	0.66		-0.89	6.00	-241.31	495.93	10.31	0.00
22.75				+	+	0.30	-0.17		7.00	-240.08	495.94	10.32	0.00
20.61				+	+	0.75			6.00	-241.41	496.14	10.52	0.00
18.73				+		0.64		-0.78	6.00	-241.50	496.31	10.69	0.00
19.73			0.19		+	-0.02	-0.18	-1.57	8.00	-239.04	496.39	10.77	0.00
20.33				+	+				5.00	-242.81	496.55	10.93	0.00
15.31			0.19	+				-1.49	6.00	-241.64	496.59	10.97	0.00

17.00			0.24	+	+				6.00	-241.78	496.87	11.25	0.00
19.89			0.19	+	+	0.16	-0.17		8.00	-239.30	496.93	11.31	0.00
17.61			0.20		+	0.55		-0.97	7.00	-240.58	496.93	11.31	0.00
17.79			0.20	+	+	0.65			7.00	-240.70	497.18	11.56	0.00
18.29			0.16	+		0.00	-0.18	-1.33	8.00	-239.45	497.23	11.61	0.00
20.25				+	+			-1.55	6.00	-242.12	497.56	11.94	0.00
22.87				+	+	0.06	-0.19	-1.33	8.00	-239.67	497.66	12.04	0.00
15.98			0.17	+		0.56		-0.84	7.00	-240.97	497.72	12.10	0.00
17.04			0.23	+	+			-1.51	7.00	-241.14	498.06	12.45	0.00
20.53				+	+	0.64		-0.80	7.00	-241.25	498.29	12.67	0.00
19.96			0.19	+	+	-0.09	-0.19	-1.42	9.00	-238.83	498.62	13.00	0.00
17.66			0.20	+	+	0.53		-0.88	8.00	-240.52	499.35	13.73	0.00
