# Supplementary material for: From eggs to bites: do ovitrap data provide reliable estimates of Aedes albopictus biting females?
Source: PeerJ. 2017 Mar 16;5:e2998. doi: 10.7717/peerj.2998 (PMC5357344; doi:10.7717/peerj.2998)
Supplement: Table S2 [file peerj-05-2998-s009.pdf]

Table S2. Result of Model-II ranking by AICc

Model Formula	AICc	RSME
HLC ~ 1 + Eggs/site/day +Site+ Temperature + Temperature^2 + Rain lag 2 +Wind	500.66	6.90
HLC ~ 1 + Eggs/site/day +Wind	500.93	9.11
HLC ~ 1 + Eggs/site/day	501.25	9.40
HLC ~ 1 + Eggs/site/day +Site+ Temperature + Temperature^2 + Rain lag 3+Wind	501.32	7.19
HLC ~ 1 + Eggs/site/day + Rain Index	501.62	9.28
HLC ~ 1 + Eggs/site/day + Temperature + Temperature^2 +Wind	501.78	8.36
HLC ~ 1 + Eggs/site/day +Site+Wind	501.89	8.77
HLC ~ 1 + Eggs/site/day + Rain lag 1 +Wind	501.92	9.31
HLC ~ 1 + Eggs/site/day + Temperature	501.93	9.49
HLC ~ 1 + Eggs/site/day + Temperature + Temperature^2	501.94	8.90
HLC ~ 1 + Eggs/site/day + Temperature lag 1+ Temperature lag 1^2 +Wind	502.03	8.23
HLC ~ 1 + Eggs/site/day + Temperature + Rain lag 1	502.03	9.86
HLC ~ 1 + Eggs/site/day + Temperature + Temperature^2 + Rain lag 3+Wind	502.11	7.70
HLC ~ 1 + Eggs/site/day + Rain lag 1	502.16	9.59
HLC ~ 1 + Eggs/site/day +Site+ Temperature + Temperature^2 +Wind	502.21	7.96
HLC ~ 1 + Eggs/site/day + Temperature + Temperature^2 + Rain Index	502.23	8.56
HLC ~ 1 + Eggs/site/day +Site	502.26	9.07
HLC ~ 1 + Eggs/site/day +Site+ Temperature lag 1+ Temperature lag 1^2 +Wind	502.42	7.84
HLC ~ 1 + Eggs/site/day + Rain Index +Wind	502.55	9.09
HLC ~ 1 + Eggs/site/day +Site+ Rain Index	502.62	8.94
HLC ~ 1 + Eggs/site/day +Site+ Temperature + Temperature^2 + Rain lag 2 + Rain lag 2^2 +Wind	502.81	6.85
HLC ~ 1 + Eggs/site/day +Site+ Temperature + Temperature^2 + Rain Index	502.82	8.17
HLC ~ 1 + Eggs/site/day + Temperature +Wind	502.83	9.20
HLC ~ 1 + Eggs/site/day +Site+ Temperature + Temperature^2	502.84	8.56
HLC ~ 1 + Eggs/site/day + Temperature lag 1 +Wind	502.88	9.16
HLC ~ 1 + Eggs/site/day + Temperature + Temperature^2 + Rain lag 2 +Wind	502.91	7.79
HLC ~ 1 + Eggs/site/day + Eggs/site/day:Site+Site+ Temperature + Temperature^2 + Rain lag 2 +Wind	502.97	6.99
HLC ~ 1 + Eggs/site/day + Temperature + Temperature^2 + Rain lag 1	502.97	9.44
HLC ~ 1 + Eggs/site/day + Temperature + Rain Index	503.01	9.34
HLC ~ 1 + Eggs/site/day +Site+ Rain lag 1 +Wind	503.02	8.98
HLC ~ 1 + Eggs/site/day +Site+ Temperature	503.11	9.17
HLC ~ 1 + Eggs/site/day + Temperature + Temperature^2 + Rain Index +Wind	503.11	8.21
HLC ~ 1 + Eggs/site/day + Temperature lag 3 +Wind	503.14	9.10
HLC ~ 1 + Eggs/site/day + Temperature lag 1+ Temperature lag 1^2	503.17	8.60
HLC ~ 1 + Eggs/site/day + Temperature lag 3	503.27	9.38
HLC ~ 1 + Eggs/site/day + Temperature lag 2 +Wind	503.27	9.12
HLC ~ 1 + Eggs/site/day + Rain lag 3+Wind	503.28	9.20
HLC ~ 1 + Eggs/site/day +Site+ Rain lag 1	503.29	9.27
HLC ~ 1 + Eggs/site/day + Rain lag 2 +Wind	503.30	9.16
HLC ~ 1 + Eggs/site/day +Site+ Temperature + Temperature^2 + Rain Index +Wind	503.37	7.77
HLC ~ 1 + Eggs/site/day + Temperature lag 1	503.39	9.43
HLC ~ 1 + Eggs/site/day + Temperature + Rain lag 1 +Wind	503.39	9.56

HLC ~ 1 + Eggs/site/day + Temperature lag 2	503.39	9.39
HLC ~ 1 + Eggs/site/day +Site+ Temperature + Rain lag 1	503.40	9.58
HLC ~ 1 + Eggs/site/day + Temperature + Temperature^2 + Rain lag 3	503.48	8.51
HLC ~ 1 + Eggs/site/day + Rain lag 3	503.49	9.51
HLC ~ 1 + Eggs/site/day +Site+ Rain Index +Wind	503.55	8.74
HLC ~ 1 + Eggs/site/day + Rain lag 2	503.55	9.46
HLC ~ 1 + Eggs/site/day + Eggs/site/day:Site+Site+ Temperature + Temperature^2 + Rain lag 3+Wind	503.57	7.19
HLC ~ 1 + Eggs/site/day + Temperature lag 1 + Rain Index	503.60	9.32
HLC ~ 1 + Eggs/site/day + Temperature + Temperature^2 + Rain lag 1 +Wind	503.64	8.86
HLC ~ 1 + Eggs/site/day +Site+ Temperature lag 1+ Temperature lag 1^2 + Rain lag 2 +Wind	503.67	7.50
HLC ~ 1 + Eggs/site/day +Site+ Temperature lag 1+ Temperature lag 1^2	503.74	8.23
HLC ~ 1 + Eggs/site/day + Rain lag 1 + Rain lag 1^2 +Wind	503.79	9.19
HLC ~ 1 + Eggs/site/day + Temperature lag 3 + Rain Index	503.80	9.26
HLC ~ 1 + Eggs/site/day + Temperature lag 1+ Temperature lag 1^2 + Rain lag 2 +Wind	503.89	7.96
HLC ~ 1 + Eggs/site/day + Temperature lag 1 + Rain lag 1 +Wind	503.93	9.38
HLC ~ 1 + Eggs/site/day + Temperature lag 2 + Rain Index	503.93	9.27
HLC ~ 1 + Eggs/site/day + Eggs/site/day:Site+Site+Wind	503.93	8.75
HLC ~ 1 + Eggs/site/day +Site+ Temperature +Wind	503.94	8.87
HLC ~ 1 + Eggs/site/day +Site+ Temperature lag 1 +Wind	503.96	8.82
HLC ~ 1 + Eggs/site/day +Site+ Temperature + Temperature^2 + Rain lag 3	503.97	8.14
HLC ~ 1 + Eggs/site/day +Site+ Temperature + Temperature^2 + Rain lag 3 + Rain lag 3^2+Wind	503.97	7.19
HLC ~ 1 + Eggs/site/day + Rain lag 1 + Rain lag 1^2	504.01	9.47
HLC ~ 1 + Eggs/site/day + Temperature lag 1+ Temperature lag 1^2 + Rain lag 3+Wind	504.01	8.03
HLC ~ 1 + Eggs/site/day +Site+ Temperature lag 1+ Temperature lag 1^2 + Rain lag 3+Wind	504.03	7.61
HLC ~ 1 + Eggs/site/day + Temperature lag 2+ Temperature lag 2^2 +Wind	504.05	8.66
HLC ~ 1 + Eggs/site/day + Temperature lag 1+ Temperature lag 1^2 + Rain lag 1 +Wind	504.05	8.50
HLC ~ 1 + Eggs/site/day + Eggs/site/day:Site+Site	504.10	9.04
HLC ~ 1 + Eggs/site/day + Temperature lag 1+ Temperature lag 1^2 + Rain Index	504.12	8.59
HLC ~ 1 + Eggs/site/day + Temperature lag 3 + Rain lag 1 +Wind	504.14	9.30
HLC ~ 1 + Eggs/site/day + Temperature lag 3 + Rain lag 1	504.15	9.57
HLC ~ 1 + Eggs/site/day + Temperature + Temperature^2 + Rain lag 2	504.15	8.79
HLC ~ 1 + Eggs/site/day +Site+ Temperature + Rain Index	504.15	9.01
HLC ~ 1 + Eggs/site/day +Site+ Temperature lag 3 +Wind	504.16	8.76
HLC ~ 1 + Eggs/site/day +Site+ Temperature + Temperature^2 + Rain lag 1 + Rain lag 1^2 +Wind	504.21	7.70
HLC ~ 1 + Eggs/site/day +Site+ Temperature + Temperature^2 + Rain lag 1	504.23	9.16
HLC ~ 1 + Eggs/site/day + Temperature lag 1+ Temperature lag 1^2 + Rain Index +Wind	504.23	8.26
HLC ~ 1 + Eggs/site/day + Temperature + Rain lag 1 + Rain lag 1^2	504.25	9.74
HLC ~ 1 + Eggs/site/day + Eggs/site/day:Site+Site+ Temperature + Temperature^2 +Wind	504.25	7.91
HLC ~ 1 + Eggs/site/day + Temperature + Rain lag 3	504.27	9.62
HLC ~ 1 + Eggs/site/day + Temperature + Rain lag 2	504.27	9.65
HLC ~ 1 + Eggs/site/day +Site+ Temperature lag 2 +Wind	504.30	8.78

HLC ~ 1 + Eggs/site/day + Temperature + Temperature^2 + Rain lag 1 + Rain lag 1^2 +Wind	504.32	8.20
HLC ~ 1 + Eggs/site/day +Site+ Temperature lag 3	504.32	9.06
HLC ~ 1 + Eggs/site/day +Site+ Rain lag 3+Wind	504.33	8.87
HLC ~ 1 + Eggs/site/day +Site+ Rain lag 2 +Wind	504.33	8.83
HLC ~ 1 + Eggs/site/day + Temperature lag 1 + Rain lag 1	504.36	9.63
HLC ~ 1 + Eggs/site/day + Temperature lag 2 + Rain lag 1 +Wind	504.37	9.32
HLC ~ 1 + Eggs/site/day + Temperature lag 3+ Temperature lag 3^2 +Wind	504.40	8.73
HLC ~ 1 + Eggs/site/day + Temperature lag 1 + Rain Index +Wind	504.42	9.14
HLC ~ 1 + Eggs/site/day + Temperature lag 2 + Rain lag 1	504.44	9.57
HLC ~ 1 + Eggs/site/day +Site+ Temperature lag 2	504.45	9.06
HLC ~ 1 + Eggs/site/day +Site+ Temperature lag 1	504.50	9.11
HLC ~ 1 + Eggs/site/day + Temperature + Temperature^2 + Rain lag 1 + Rain lag 1^2	504.52	9.02
HLC ~ 1 + Eggs/site/day +Site+ Temperature + Temperature^2 + Rain lag 1 +Wind	504.53	8.53
HLC ~ 1 + Eggs/site/day + Temperature lag 2+ Temperature lag 2^2	504.54	9.02
HLC ~ 1 + Eggs/site/day + Eggs/site/day:Site+Site+ Temperature lag 1+ Temperature lag 1^2 +Wind	504.58	7.81
HLC ~ 1 + Eggs/site/day +Site+ Rain lag 3	504.59	9.20
HLC ~ 1 + Eggs/site/day + Temperature + Rain Index +Wind	504.61	9.16
HLC ~ 1 + Eggs/site/day + Eggs/site/day:Site+Site+ Rain Index	504.64	8.92
HLC ~ 1 + Eggs/site/day +Site+ Rain lag 2	504.64	9.14
HLC ~ 1 + Eggs/site/day + Temperature + Temperature^2 + Rain lag 3 + Rain lag 3^2+Wind	504.67	7.69
HLC ~ 1 + Eggs/site/day +Site+ Temperature + Temperature^2 + Rain lag 2	504.67	8.46
HLC ~ 1 + Eggs/site/day +Site+ Temperature + Rain lag 1 +Wind	504.70	9.25
HLC ~ 1 + Eggs/site/day +Site+ Temperature lag 1 + Rain Index	504.70	8.98
HLC ~ 1 + Eggs/site/day +Site+ Temperature lag 1+ Temperature lag 1^2 + Rain Index +Wind	504.71	7.87
HLC ~ 1 + Eggs/site/day +Site+ Temperature lag 1+ Temperature lag 1^2 + Rain lag 1 +Wind	504.72	8.14
HLC ~ 1 + Eggs/site/day + Eggs/site/day:Site+Site+ Temperature + Temperature^2	504.72	8.51
HLC ~ 1 + Eggs/site/day +Site+ Temperature lag 1+ Temperature lag 1^2 + Rain Index	504.73	8.21
HLC ~ 1 + Eggs/site/day +Site+ Temperature lag 2+ Temperature lag 2^2 +Wind	504.83	8.32
HLC ~ 1 + Eggs/site/day +Site+ Temperature lag 3 + Rain Index	504.85	8.92
HLC ~ 1 + Eggs/site/day + Temperature lag 3+ Temperature lag 3^2	504.85	9.08
HLC ~ 1 + Eggs/site/day + Temperature lag 3 + Rain Index +Wind	504.86	9.08
HLC ~ 1 + Eggs/site/day + Eggs/site/day:Site+Site+ Temperature + Temperature^2 + Rain Index	504.88	8.13
HLC ~ 1 + Eggs/site/day +Site+ Rain lag 1 + Rain lag 1^2 +Wind	504.89	8.84
HLC ~ 1 + Eggs/site/day + Temperature lag 1+ Temperature lag 1^2 + Rain lag 1	504.98	8.88
HLC ~ 1 + Eggs/site/day + Temperature lag 2 + Rain Index +Wind	504.98	9.10
HLC ~ 1 + Eggs/site/day +Site+ Temperature lag 2 + Rain Index	504.98	8.93
HLC ~ 1 + Eggs/site/day + Eggs/site/day:Site+Site+ Temperature	505.05	9.14
HLC ~ 1 + Eggs/site/day + Temperature + Temperature^2 + Rain lag 2 + Rain lag 2^2 +Wind	505.13	7.69
HLC ~ 1 + Eggs/site/day +Site+ Rain lag 1 + Rain lag 1^2	505.14	9.14
HLC ~ 1 + Eggs/site/day +Site+ Temperature lag 1 + Rain lag 1 +Wind	505.16	9.05

HLC ~ 1 + Eggs/site/day +Site+ Temperature lag 1+ Temperature lag 1^2 + Rain lag 3 + Rain lag 3^2+Wind	505.18	7.34
HLC ~ 1 + Eggs/site/day +Site+ Temperature lag 3+ Temperature lag 3^2 +Wind	505.21	8.39
HLC ~ 1 + Eggs/site/day + Eggs/site/day:Site+Site+ Rain lag 1 +Wind	505.25	8.94
HLC ~ 1 + Eggs/site/day + Temperature + Rain lag 3+Wind	505.26	9.30
HLC ~ 1 + Eggs/site/day + Temperature + Rain lag 2 +Wind	505.27	9.31
HLC ~ 1 + Eggs/site/day + Temperature lag 1+ Temperature lag 1^2 + Rain lag 2	505.27	8.45
HLC ~ 1 + Eggs/site/day +Site+ Temperature lag 3 + Rain lag 1 +Wind	505.30	8.98
HLC ~ 1 + Eggs/site/day + Eggs/site/day:Site+Site+ Rain lag 1	505.33	9.23
HLC ~ 1 + Eggs/site/day + Temperature lag 1 + Rain lag 2 +Wind	505.33	9.23
HLC ~ 1 + Eggs/site/day + Temperature lag 1 + Rain lag 3+Wind	505.33	9.21
HLC ~ 1 + Eggs/site/day +Site+ Temperature lag 3 + Rain lag 1	505.33	9.27
HLC ~ 1 + Eggs/site/day + Eggs/site/day:Site+Site+ Temperature + Temperature^2 + Rain lag 2 + Rain lag 2^2 +Wind	505.33	6.92
HLC ~ 1 + Eggs/site/day + Temperature lag 3+ Temperature lag 3^2 + Rain Index	505.36	8.93
HLC ~ 1 + Eggs/site/day + Rain lag 3 + Rain lag 3^2+Wind	505.40	9.10
HLC ~ 1 + Eggs/site/day + Temperature lag 2+ Temperature lag 2^2 + Rain Index	505.41	8.93
HLC ~ 1 + Eggs/site/day +Site+ Temperature lag 2+ Temperature lag 2^2	505.46	8.70
HLC ~ 1 + Eggs/site/day + Temperature lag 1+ Temperature lag 1^2 + Rain lag 1 + Rain lag 1^2 +Wind	505.47	8.25
HLC ~ 1 + Eggs/site/day + Temperature lag 1+ Temperature lag 1^2 + Rain lag 3	505.48	8.57
HLC ~ 1 + Eggs/site/day +Site+ Temperature + Temperature^2 + Rain lag 1 + Rain lag 1^2	505.49	8.71
HLC ~ 1 + Eggs/site/day + Temperature lag 1+ Temperature lag 1^2 + Rain lag 3 + Rain lag 3^2+Wind	505.53	7.80
HLC ~ 1 + Eggs/site/day +Site+ Temperature lag 2 + Rain lag 1 +Wind	505.54	9.00
HLC ~ 1 + Eggs/site/day +Site+ Temperature + Rain lag 3	505.54	9.33
HLC ~ 1 + Eggs/site/day + Temperature + Rain lag 1 + Rain lag 1^2 +Wind	505.54	9.40
HLC ~ 1 + Eggs/site/day +Site+ Temperature + Rain lag 2	505.55	9.36
HLC ~ 1 + Eggs/site/day + Eggs/site/day:Site+Site+ Temperature + Temperature^2 + Rain Index +Wind	505.55	7.73
HLC ~ 1 + Eggs/site/day +Site+ Temperature lag 1 + Rain Index +Wind	505.56	8.80
HLC ~ 1 + Eggs/site/day + Temperature lag 3 + Rain lag 3+Wind	505.57	9.22
HLC ~ 1 + Eggs/site/day + Temperature lag 3 + Rain lag 3	505.58	9.52
HLC ~ 1 + Eggs/site/day +Site+ Temperature lag 1+ Temperature lag 1^2 + Rain lag 2	505.58	8.06
HLC ~ 1 + Eggs/site/day +Site+ Temperature lag 1 + Rain lag 1	505.59	9.32
HLC ~ 1 + Eggs/site/day + Eggs/site/day:Site+Site+ Temperature + Rain lag 1	505.59	9.53
HLC ~ 1 + Eggs/site/day + Temperature lag 3 + Rain lag 2 +Wind	505.60	9.17
HLC ~ 1 + Eggs/site/day +Site+ Temperature lag 2 + Rain lag 1	505.62	9.26
HLC ~ 1 + Eggs/site/day +Site+ Temperature + Rain lag 1 + Rain lag 1^2	505.62	9.45
HLC ~ 1 + Eggs/site/day + Temperature lag 3 + Rain lag 2	505.64	9.45
HLC ~ 1 + Eggs/site/day + Eggs/site/day:Site+Site+ Temperature lag 1+ Temperature lag 1^2	505.67	8.19
HLC ~ 1 + Eggs/site/day + Temperature lag 2 + Rain lag 3	505.69	9.52
HLC ~ 1 + Eggs/site/day + Temperature lag 2 + Rain lag 3+Wind	505.70	9.22
HLC ~ 1 + Eggs/site/day + Eggs/site/day:Site+Site+ Rain Index +Wind	505.71	8.73
HLC ~ 1 + Eggs/site/day + Rain lag 2 + Rain lag 2^2	505.72	9.40
HLC ~ 1 + Eggs/site/day + Temperature lag 2 + Rain lag 2 +Wind	505.72	9.18

HLC ~ 1 + Eggs/site/day + Rain lag 3 + Rain lag 3^2	505.73	9.45
HLC ~ 1 + Eggs/site/day + Temperature lag 1 + Rain lag 3	505.73	9.52
HLC ~ 1 + Eggs/site/day + Rain lag 2 + Rain lag 2^2 +Wind	505.74	9.15
HLC ~ 1 + Eggs/site/day +Site+ Temperature + Rain Index +Wind	505.75	8.82
HLC ~ 1 + Eggs/site/day + Temperature lag 1 + Rain lag 2	505.76	9.51
HLC ~ 1 + Eggs/site/day + Temperature lag 2 + Rain lag 2	505.77	9.44
HLC ~ 1 + Eggs/site/day +Site+ Temperature lag 3+ Temperature lag 3^2	505.77	8.77
HLC ~ 1 + Eggs/site/day +Site+ Temperature lag 1+ Temperature lag 1^2 + Rain lag 1	505.79	8.55
HLC ~ 1 + Eggs/site/day +Site+ Temperature + Temperature^2 + Rain lag 2 + Rain lag 2^2	505.82	8.08
HLC ~ 1 + Eggs/site/day + Temperature lag 2+ Temperature lag 2^2 + Rain lag 1 +Wind	505.85	8.92
HLC ~ 1 + Eggs/site/day + Temperature lag 1 + Rain lag 1 + Rain lag 1^2 +Wind	505.85	9.23
HLC ~ 1 + Eggs/site/day + Temperature + Temperature^2 + Rain lag 2 + Rain lag 2^2	505.85	8.50
HLC ~ 1 + Eggs/site/day +Site+ Temperature lag 3 + Rain Index +Wind	505.92	8.74
HLC ~ 1 + Eggs/site/day +Site+ Temperature lag 1+ Temperature lag 1^2 + Rain lag 1 + Rain lag 1^2 +Wind	505.96	7.85
HLC ~ 1 + Eggs/site/day +Site+ Temperature lag 1+ Temperature lag 1^2 + Rain lag 3	505.96	8.21
HLC ~ 1 + Eggs/site/day + Temperature + Temperature^2 + Rain lag 3 + Rain lag 3^2	505.96	8.48
HLC ~ 1 + Eggs/site/day + Eggs/site/day:Site+Site+ Temperature + Temperature^2 + Rain lag 3	506.00	8.12
HLC ~ 1 + Eggs/site/day + Eggs/site/day:Site+Site+ Temperature lag 1+ Temperature lag 1^2 + Rain lag 2 +Wind	506.01	7.50
HLC ~ 1 + Eggs/site/day +Site+ Temperature lag 2 + Rain Index +Wind	506.05	8.75
HLC ~ 1 + Eggs/site/day + Eggs/site/day:Site+Site+ Temperature +Wind	506.05	8.85
HLC ~ 1 + Eggs/site/day + Eggs/site/day:Site+Site+ Temperature lag 1 +Wind	506.09	8.81
HLC ~ 1 + Eggs/site/day + Temperature lag 2+ Temperature lag 2^2 + Rain Index +Wind	506.11	8.66
HLC ~ 1 + Eggs/site/day + Temperature lag 3 + Rain lag 1 + Rain lag 1^2	506.13	9.47
HLC ~ 1 + Eggs/site/day + Temperature lag 3 + Rain lag 1 + Rain lag 1^2 +Wind	506.14	9.19
HLC ~ 1 + Eggs/site/day + Temperature lag 2+ Temperature lag 2^2 + Rain lag 1	506.15	9.27
HLC ~ 1 + Eggs/site/day + Temperature lag 3+ Temperature lag 3^2 + Rain lag 1 +Wind	506.15	9.07
HLC ~ 1 + Eggs/site/day + Temperature lag 3+ Temperature lag 3^2 + Rain Index +Wind	506.20	8.70
HLC ~ 1 + Eggs/site/day + Eggs/site/day:Site+Site+ Temperature lag 3	506.21	9.02
HLC ~ 1 + Eggs/site/day +Site+ Temperature lag 3+ Temperature lag 3^2 + Rain Index	506.23	8.59
HLC ~ 1 + Eggs/site/day + Eggs/site/day:Site+Site+ Temperature lag 3 +Wind	506.26	8.74
HLC ~ 1 + Eggs/site/day + Eggs/site/day:Site+Site+ Temperature + Rain Index	506.26	8.99
HLC ~ 1 + Eggs/site/day + Temperature lag 1 + Rain lag 1 + Rain lag 1^2	506.27	9.50
HLC ~ 1 + Eggs/site/day + Eggs/site/day:Site+Site+ Temperature + Temperature^2 + Rain lag 3 + Rain lag 3^2+Wind	506.29	7.19
HLC ~ 1 + Eggs/site/day +Site+ Temperature lag 1+ Temperature lag 1^2 + Rain lag 2 + Rain lag 2^2 +Wind	506.29	7.49
HLC ~ 1 + Eggs/site/day + Temperature lag 2 + Rain lag 1 + Rain lag 1^2 +Wind	506.32	9.21
HLC ~ 1 + Eggs/site/day +Site+ Temperature lag 2+ Temperature lag 2^2 + Rain Index	506.32	8.60
HLC ~ 1 + Eggs/site/day + Eggs/site/day:Site+Site+ Temperature lag 2	506.33	9.03
HLC ~ 1 + Eggs/site/day + Temperature lag 3+ Temperature lag 3^2 + Rain lag 1	506.33	9.45
HLC ~ 1 + Eggs/site/day + Eggs/site/day:Site+Site+ Temperature lag 1+ Temperature lag 1^2 + Rain lag 3+Wind	506.35	7.61
HLC ~ 1 + Eggs/site/day + Eggs/site/day:Site+Site+ Temperature + Temperature^2 + Rain lag 1	506.37	9.07

HLC ~ 1 + Temperature	506.37	10.35
HLC ~ 1 + Eggs/site/day + Temperature lag 2 + Rain lag 1 + Rain lag 1^2	506.38	9.46
HLC ~ 1 + Eggs/site/day + Eggs/site/day:Site+Site+ Temperature lag 2 +Wind	506.40	8.76
HLC ~ 1 + Eggs/site/day + Eggs/site/day:Site+Site+ Temperature lag 1	506.40	9.08
HLC ~ 1 + Eggs/site/day + Temperature lag 2+ Temperature lag 2^2 + Rain lag 2 +Wind	506.41	8.60
HLC ~ 1 + Eggs/site/day + Eggs/site/day:Site+Site+ Rain lag 3+Wind	506.44	8.86
HLC ~ 1 + Eggs/site/day + Temperature lag 1+ Temperature lag 1^2 + Rain lag 2 + Rain lag 2^2 +Wind	506.46	7.95
HLC ~ 1 + Eggs/site/day + Eggs/site/day:Site+Site+ Rain lag 2 +Wind	506.46	8.82
HLC ~ 1 + Eggs/site/day +Site+ Temperature + Rain lag 3+Wind	506.46	8.99
HLC ~ 1 + Eggs/site/day + Temperature + Rain lag 3 + Rain lag 3^2	506.46	9.57
HLC ~ 1 + Eggs/site/day +Site+ Temperature + Rain lag 2 +Wind	506.46	9.00
HLC ~ 1 + Eggs/site/day +Site+ Rain lag 3 + Rain lag 3^2+Wind	506.47	8.76
HLC ~ 1 + Eggs/site/day + Eggs/site/day:Site+Site+ Rain lag 3	506.49	9.17
HLC ~ 1 + Eggs/site/day +Site+ Temperature lag 1 + Rain lag 2 +Wind	506.49	8.92
HLC ~ 1 + Eggs/site/day + Temperature lag 1+ Temperature lag 1^2 + Rain lag 1 + Rain lag 1^2	506.49	8.64
HLC ~ 1 + Eggs/site/day +Site+ Temperature lag 1 + Rain lag 3+Wind	506.49	8.89
HLC ~ 1 + Eggs/site/day +Site+ Temperature + Temperature^2 + Rain lag 3 + Rain lag 3^2	506.50	8.11
HLC ~ 1 + Eggs/site/day + Temperature lag 2+ Temperature lag 2^2 + Rain lag 3+Wind	506.53	8.70
HLC ~ 1 + Eggs/site/day + Eggs/site/day:Site+Site+ Rain lag 2	506.55	9.12
HLC ~ 1 + Eggs/site/day + Temperature + Rain lag 2 + Rain lag 2^2	506.55	9.57
HLC ~ 1 + Temperature + Temperature^2	506.58	9.77
HLC ~ 1 + Eggs/site/day + Eggs/site/day:Site+Site+ Temperature + Temperature^2 + Rain lag 1 + Rain lag 1^2 +Wind	506.59	7.64
HLC ~ 1 + Eggs/site/day + Temperature lag 3+ Temperature lag 3^2 + Rain lag 2 +Wind	506.66	8.65
HLC ~ 1 + Eggs/site/day +Site+ Temperature lag 3 + Rain lag 2 +Wind	506.68	8.85
HLC ~ 1 + Eggs/site/day +Site+ Temperature lag 3 + Rain lag 3+Wind	506.68	8.91
HLC ~ 1 + Eggs/site/day + Temperature lag 3+ Temperature lag 3^2 + Rain lag 3+Wind	506.70	8.69
HLC ~ 1 + Eggs/site/day + Eggs/site/day:Site+Site+ Temperature + Temperature^2 + Rain lag 2	506.70	8.43
HLC ~ 1 + Eggs/site/day +Site+ Temperature lag 3 + Rain lag 3	506.73	9.23
HLC ~ 1 + Eggs/site/day + Eggs/site/day:Site+Site+ Temperature + Temperature^2 + Rain lag 1 +Wind	506.74	8.44
HLC ~ 1 + Eggs/site/day +Site+ Temperature lag 3 + Rain lag 2	506.77	9.16
HLC ~ 1 + Eggs/site/day + Eggs/site/day:Site+Site+ Temperature lag 1 + Rain Index	506.80	8.97
HLC ~ 1 + Eggs/site/day +Site+ Temperature lag 2 + Rain lag 2 +Wind	506.81	8.86
HLC ~ 1 + Eggs/site/day +Site+ Temperature lag 2 + Rain lag 3+Wind	506.82	8.91
HLC ~ 1 + Eggs/site/day +Site+ Rain lag 2 + Rain lag 2^2	506.83	9.07
HLC ~ 1 + Eggs/site/day + Eggs/site/day:Site+Site+ Temperature lag 2+ Temperature lag 2^2 +Wind	506.83	8.26
HLC ~ 1 + Eggs/site/day +Site+ Temperature lag 2+ Temperature lag 2^2 + Rain lag 1 +Wind	506.84	8.60
HLC ~ 1 + Eggs/site/day +Site+ Rain lag 2 + Rain lag 2^2 +Wind	506.84	8.82
HLC ~ 1 + Eggs/site/day +Site+ Temperature + Rain lag 1 + Rain lag 1^2 +Wind	506.85	9.09
HLC ~ 1 + Eggs/site/day +Site+ Temperature lag 2 + Rain lag 3	506.85	9.22

HLC ~ 1 + Eggs/site/day + Eggs/site/day:Site+Site+ Temperature lag 1+ Temperature lag 1^2 + Rain Index	506.86	8.18
HLC ~ 1 + Eggs/site/day +Site+ Rain lag 3 + Rain lag 3^2	506.88	9.14
HLC ~ 1 + Eggs/site/day +Site+ Temperature lag 2 + Rain lag 2	506.89	9.14
HLC ~ 1 + Eggs/site/day + Temperature lag 2+ Temperature lag 2^2 + Rain lag 2	506.91	9.00
HLC ~ 1 + Eggs/site/day + Eggs/site/day:Site+Site+ Temperature lag 3 + Rain Index	506.92	8.90
HLC ~ 1 + Eggs/site/day +Site+ Temperature lag 1 + Rain lag 3	506.93	9.22
HLC ~ 1 + Eggs/site/day +Site+ Temperature lag 1 + Rain lag 2	506.95	9.20
HLC ~ 1 + Eggs/site/day +Site+ Temperature lag 2+ Temperature lag 2^2 + Rain Index +Wind	506.95	8.31
HLC ~ 1 + Eggs/site/day + Temperature lag 2+ Temperature lag 2^2 + Rain lag 3	506.99	9.13
HLC ~ 1 + Eggs/site/day + Eggs/site/day:Site+Site+ Temperature lag 1+ Temperature lag 1^2 + Rain Index +Wind	506.99	7.83
HLC ~ 1 + Eggs/site/day + Eggs/site/day:Site+Site+ Temperature lag 1+ Temperature lag 1^2 + Rain lag 1 +Wind	507.01	8.08
HLC ~ 1 + Eggs/site/day + Eggs/site/day:Site+Site+ Temperature + Rain lag 1 +Wind	507.03	9.21
HLC ~ 1 + Eggs/site/day +Site+ Temperature lag 3+ Temperature lag 3^2 + Rain Index +Wind	507.03	8.34
HLC ~ 1 + Eggs/site/day + Eggs/site/day:Site+Site+ Temperature lag 2 + Rain Index	507.06	8.91
HLC ~ 1 + Eggs/site/day +Site+ Temperature lag 1 + Rain lag 1 + Rain lag 1^2 +Wind	507.07	8.90
HLC ~ 1 + Eggs/site/day +Site+ Temperature lag 2+ Temperature lag 2^2 + Rain lag 2 +Wind	507.08	8.26
HLC ~ 1 + Eggs/site/day +Site+ Temperature lag 1+ Temperature lag 1^2 + Rain lag 1 + Rain lag 1^2	507.16	8.27
HLC ~ 1 + Eggs/site/day + Temperature lag 3+ Temperature lag 3^2 + Rain lag 2	507.19	9.09
HLC ~ 1 + Eggs/site/day +Site+ Temperature lag 3+ Temperature lag 3^2 + Rain lag 1 +Wind	507.20	8.76
HLC ~ 1 + Eggs/site/day + Eggs/site/day:Site+Site+ Temperature lag 2+ Temperature lag 2^2	507.23	8.63
HLC ~ 1 + Eggs/site/day +Site+ Temperature lag 2+ Temperature lag 2^2 + Rain lag 1	507.25	8.97
HLC ~ 1 + Eggs/site/day + Eggs/site/day:Site+Site+ Temperature lag 3+ Temperature lag 3^2 +Wind	507.25	8.33
HLC ~ 1 + Eggs/site/day +Site+ Temperature lag 3+ Temperature lag 3^2 + Rain lag 2 +Wind	507.26	8.32
HLC ~ 1 + Eggs/site/day + Temperature lag 3+ Temperature lag 3^2 + Rain lag 3	507.27	9.20
HLC ~ 1 + Temperature + Rain lag 1	507.27	10.67
HLC ~ 1 + Eggs/site/day + Eggs/site/day:Site+Site+ Rain lag 1 + Rain lag 1^2 +Wind	507.27	8.82
HLC ~ 1 + Eggs/site/day +Site+ Temperature lag 3 + Rain lag 1 + Rain lag 1^2 +Wind	507.31	8.86
HLC ~ 1 + Eggs/site/day +Site+ Temperature lag 2+ Temperature lag 2^2 + Rain lag 3+Wind	507.31	8.37
HLC ~ 1 + Eggs/site/day +Site+ Temperature lag 3 + Rain lag 1 + Rain lag 1^2	507.33	9.15
HLC ~ 1 + Eggs/site/day + Eggs/site/day:Site+Site+ Rain lag 1 + Rain lag 1^2	507.33	9.11
HLC ~ 1	507.35	10.44
HLC ~ 1 + Eggs/site/day +Site+ Temperature lag 3+ Temperature lag 3^2 + Rain lag 3+Wind	507.39	8.37
HLC ~ 1 + Eggs/site/day + Temperature lag 2+ Temperature lag 2^2 + Rain lag 1 + Rain lag 1^2 +Wind	507.41	8.68
HLC ~ 1 + Eggs/site/day + Temperature + Rain lag 3 + Rain lag 3^2+Wind	507.42	9.21
HLC ~ 1 + Eggs/site/day + Eggs/site/day:Site+Site+ Temperature lag 3 + Rain lag 1	507.43	9.21

HLC ~ 1 + Eggs/site/day + Temperature lag 1+ Temperature lag 1^2 + Rain lag 2 + Rain lag 2^2	507.43	8.37
HLC ~ 1 + Eggs/site/day + Temperature lag 1+ Temperature lag 1^2 + Rain lag 3 + Rain lag 3^2	507.47	8.41
HLC ~ 1 + Eggs/site/day +Site+ Temperature lag 3+ Temperature lag 3^2 + Rain lag 1	507.47	9.18
HLC ~ 1 + Eggs/site/day + Eggs/site/day:Site+Site+ Temperature lag 1 + Rain lag 1 +Wind	507.47	9.02
HLC ~ 1 + Eggs/site/day +Site+ Temperature lag 2 + Rain lag 1 + Rain lag 1^2 +Wind	507.49	8.87
HLC ~ 1 + Eggs/site/day +Site+ Temperature lag 1 + Rain lag 1 + Rain lag 1^2	507.50	9.18
HLC ~ 1 + Eggs/site/day + Eggs/site/day:Site+Site+ Temperature + Rain lag 3	507.55	9.31
HLC ~ 1 + Eggs/site/day + Eggs/site/day:Site+Site+ Temperature + Rain lag 2	507.56	9.34
HLC ~ 1 + Eggs/site/day +Site+ Temperature lag 2 + Rain lag 1 + Rain lag 1^2	507.57	9.14
HLC ~ 1 + Eggs/site/day + Eggs/site/day:Site+Site+ Temperature lag 3 + Rain lag 1 +Wind	507.59	8.94
HLC ~ 1 + Eggs/site/day + Eggs/site/day:Site+Site+ Temperature lag 3+ Temperature lag 3^2	507.60	8.69
HLC ~ 1 + Eggs/site/day + Eggs/site/day:Site+Site+ Temperature lag 1+ Temperature lag 1^2 + Rain lag 3 + Rain lag 3^2+Wind	507.63	7.34
HLC ~ 1 + Eggs/site/day + Temperature lag 1 + Rain lag 3 + Rain lag 3^2+Wind	507.66	9.14
HLC ~ 1 + Eggs/site/day + Eggs/site/day:Site+Site+ Temperature lag 1+ Temperature lag 1^2 + Rain lag 2	507.66	8.05
HLC ~ 1 + Temperature + Temperature^2 + Rain lag 3	507.67	9.16
HLC ~ 1 + Eggs/site/day +Site+ Temperature lag 1+ Temperature lag 1^2 + Rain lag 2 + Rain lag 2^2	507.70	7.97
HLC ~ 1 + Eggs/site/day + Eggs/site/day:Site+Site+ Temperature lag 1 + Rain lag 1	507.71	9.28
HLC ~ 1 + Eggs/site/day + Eggs/site/day:Site+Site+ Temperature lag 2 + Rain lag 1	507.72	9.21
HLC ~ 1 + Eggs/site/day + Temperature lag 3 + Rain lag 3 + Rain lag 3^2+Wind	507.72	9.11
HLC ~ 1 + Eggs/site/day + Eggs/site/day:Site+Site+ Temperature + Temperature^2 + Rain lag 1 + Rain lag 1^2	507.77	8.64
HLC ~ 1 + Eggs/site/day + Temperature + Rain lag 2 + Rain lag 2^2 +Wind	507.78	9.29
HLC ~ 1 +Wind	507.79	10.20
HLC ~ 1 + Eggs/site/day +Site+ Temperature + Rain lag 3 + Rain lag 3^2	507.80	9.28
HLC ~ 1 + Eggs/site/day + Eggs/site/day:Site+Site+ Temperature lag 1 + Rain Index +Wind	507.81	8.79
HLC ~ 1 + Eggs/site/day +Site+ Temperature lag 1+ Temperature lag 1^2 + Rain lag 3 + Rain lag 3^2	507.81	8.03
HLC ~ 1 + Eggs/site/day +Site+ Temperature lag 2+ Temperature lag 2^2 + Rain lag 2	507.82	8.70
HLC ~ 1 + Eggs/site/day + Temperature lag 3+ Temperature lag 3^2 + Rain lag 1 + Rain lag 1^2 +Wind	507.82	8.81
HLC ~ 1 + Eggs/site/day + Temperature lag 2+ Temperature lag 2^2 + Rain lag 1 + Rain lag 1^2	507.83	9.05
HLC ~ 1 + Eggs/site/day + Temperature lag 1 + Rain lag 2 + Rain lag 2^2 +Wind	507.83	9.22
HLC ~ 1 + Eggs/site/day + Eggs/site/day:Site+Site+ Temperature lag 2 + Rain lag 1 +Wind	507.84	8.96
HLC ~ 1 + Eggs/site/day + Temperature lag 3 + Rain lag 3 + Rain lag 3^2	507.84	9.45
HLC ~ 1 + Eggs/site/day +Site+ Temperature + Rain lag 2 + Rain lag 2^2	507.85	9.27
HLC ~ 1 + Eggs/site/day + Temperature lag 2 + Rain lag 3 + Rain lag 3^2+Wind	507.88	9.12
HLC ~ 1 + Eggs/site/day + Eggs/site/day:Site+Site+ Temperature lag 1+ Temperature lag 1^2 + Rain lag 1	507.89	8.48
HLC ~ 1 +Site+ Temperature	507.89	10.12
HLC ~ 1 + Eggs/site/day + Temperature lag 3 + Rain lag 2 + Rain lag 2^2	507.90	9.40
HLC ~ 1 + Temperature + Temperature^2 +Wind	507.91	9.47

HLC ~ 1 + Eggs/site/day + Eggs/site/day:Site+Site+ Temperature + Rain lag 1 + Rain lag 1^2	507.94	9.41
HLC ~ 1 + Eggs/site/day + Temperature lag 1 + Rain lag 2 + Rain lag 2^2	507.95	9.44
HLC ~ 1 + Eggs/site/day +Site+ Temperature lag 2+ Temperature lag 2^2 + Rain lag 3	507.98	8.84
HLC ~ 1 + Eggs/site/day + Temperature lag 2 + Rain lag 3 + Rain lag 3^2	507.98	9.46
HLC ~ 1 + Eggs/site/day + Eggs/site/day:Site+Site+ Temperature + Rain Index +Wind	507.99	8.81
HLC ~ 1 +Site+ Temperature + Temperature^2	508.00	9.54
HLC ~ 1 + Eggs/site/day + Temperature lag 2 + Rain lag 2 + Rain lag 2^2	508.01	9.39
HLC ~ 1 + Eggs/site/day + Eggs/site/day:Site+Site+ Temperature lag 1+ Temperature lag 1^2 + Rain lag 3	508.02	8.19
HLC ~ 1 + Eggs/site/day +Site+ Temperature lag 3+ Temperature lag 3^2 + Rain lag 2	508.03	8.81
HLC ~ 1 + Temperature + Temperature^2 + Rain lag 3+Wind	508.08	8.58
HLC ~ 1 + Eggs/site/day + Temperature lag 1 + Rain lag 3 + Rain lag 3^2	508.10	9.48
HLC ~ 1 + Temperature + Temperature^2 + Rain lag 1	508.11	10.19
HLC ~ 1 + Eggs/site/day + Temperature lag 3 + Rain lag 2 + Rain lag 2^2 +Wind	508.12	9.16
HLC ~ 1 + Eggs/site/day + Temperature lag 3+ Temperature lag 3^2 + Rain lag 1 + Rain lag 1^2	508.12	9.21
HLC ~ 1 + Eggs/site/day + Eggs/site/day:Site+Site+ Temperature lag 3 + Rain Index +Wind	508.14	8.71
HLC ~ 1 + Temperature +Wind	508.16	10.21
HLC ~ 1 + Eggs/site/day + Eggs/site/day:Site+Site+ Temperature + Temperature^2 + Rain lag 2 + Rain lag 2^2	508.18	8.11
HLC ~ 1 + Eggs/site/day +Site+ Temperature lag 3+ Temperature lag 3^2 + Rain lag 3	508.19	8.95
HLC ~ 1 + Eggs/site/day + Temperature lag 2 + Rain lag 2 + Rain lag 2^2 +Wind	508.24	9.17
HLC ~ 1 + Eggs/site/day + Eggs/site/day:Site+Site+ Temperature lag 3+ Temperature lag 3^2 + Rain Index	508.26	8.53
HLC ~ 1 + Eggs/site/day +Site+ Temperature lag 2+ Temperature lag 2^2 + Rain lag 1 + Rain lag 1^2 +Wind	508.28	8.33
HLC ~ 1 + Eggs/site/day + Eggs/site/day:Site+Site+ Temperature lag 2 + Rain Index +Wind	508.28	8.73
HLC ~ 1 + Temperature + Temperature^2 + Rain Index	508.31	9.64
HLC ~ 1 + Eggs/site/day + Eggs/site/day:Site+Site+ Temperature lag 2+ Temperature lag 2^2 + Rain Index	508.33	8.54
HLC ~ 1 + Temperature + Rain Index	508.37	10.31
HLC ~ 1 + Temperature lag 1 +Wind	508.37	10.12
HLC ~ 1 + Eggs/site/day + Eggs/site/day:Site+Site+ Temperature lag 1+ Temperature lag 1^2 + Rain lag 1 + Rain lag 1^2 +Wind	508.46	7.82
HLC ~ 1 + Temperature lag 1	508.46	10.37
HLC ~ 1 + Eggs/site/day + Eggs/site/day:Site+Site+ Temperature + Temperature^2 + Rain lag 3 + Rain lag 3^2	508.57	8.08
HLC ~ 1 + Eggs/site/day + Temperature lag 2+ Temperature lag 2^2 + Rain lag 2 + Rain lag 2^2	508.65	8.78
HLC ~ 1 + Eggs/site/day +Site+ Temperature + Rain lag 3 + Rain lag 3^2+Wind	508.65	8.90
HLC ~ 1 + Eggs/site/day + Eggs/site/day:Site+Site+ Temperature + Rain lag 3+Wind	508.65	8.99
HLC ~ 1 + Temperature + Rain lag 2	508.66	10.49
HLC ~ 1 + Eggs/site/day + Eggs/site/day:Site+Site+ Temperature + Rain lag 2 +Wind	508.66	9.00
HLC ~ 1 + Eggs/site/day + Temperature lag 2+ Temperature lag 2^2 + Rain lag 2 + Rain lag 2^2 +Wind	508.68	8.48
HLC ~ 1 + Temperature + Rain lag 3	508.68	10.41
HLC ~ 1 + Eggs/site/day + Eggs/site/day:Site+Site+ Temperature lag 3 + Rain lag 3	508.68	9.19

HLC ~ 1 + Eggs/site/day + Eggs/site/day:Site+Site+ Temperature lag 1 + Rain lag 2 +Wind	508.69	8.91
HLC ~ 1 + Eggs/site/day + Eggs/site/day:Site+Site+ Temperature lag 1 + Rain lag 3+Wind	508.69	8.88
HLC ~ 1 + Eggs/site/day + Eggs/site/day:Site+Site+ Rain lag 3 + Rain lag 3^2+Wind	508.72	8.76
HLC ~ 1 + Eggs/site/day + Eggs/site/day:Site+Site+ Temperature lag 3 + Rain lag 2	508.74	9.12
HLC ~ 1 + Eggs/site/day +Site+ Temperature lag 3+ Temperature lag 3^2 + Rain lag 1 + Rain lag 1^2 +Wind	508.75	8.48
HLC ~ 1 + Eggs/site/day + Eggs/site/day:Site+Site+ Temperature lag 1+ Temperature lag 1^2 + Rain lag 2 + Rain lag 2^2 +Wind	508.76	7.50
HLC ~ 1 + Temperature + Temperature^2 + Rain lag 2	508.78	9.66
HLC ~ 1 + Eggs/site/day + Eggs/site/day:Site+Site+ Temperature lag 2 + Rain lag 3	508.79	9.19
HLC ~ 1 +Site	508.81	10.22
HLC ~ 1 + Rain Index	508.81	10.40
HLC ~ 1 + Eggs/site/day +Site+ Temperature lag 3 + Rain lag 3 + Rain lag 3^2+Wind	508.83	8.79
HLC ~ 1 +Site+ Temperature + Temperature^2 + Rain lag 3	508.83	8.94
HLC ~ 1 + Eggs/site/day +Site+ Temperature lag 2+ Temperature lag 2^2 + Rain lag 1 + Rain lag 1^2	508.85	8.73
HLC ~ 1 + Eggs/site/day + Eggs/site/day:Site+Site+ Temperature lag 3 + Rain lag 3+Wind	508.85	8.88
HLC ~ 1 + Eggs/site/day +Site+ Temperature lag 1 + Rain lag 3 + Rain lag 3^2+Wind	508.85	8.82
HLC ~ 1 + Eggs/site/day + Eggs/site/day:Site+Site+ Temperature lag 2 + Rain lag 2	508.86	9.11
HLC ~ 1 + Eggs/site/day + Eggs/site/day:Site+Site+ Temperature lag 3 + Rain lag 2 +Wind	508.86	8.83
HLC ~ 1 +Site+ Temperature + Temperature^2 + Rain lag 3+Wind	508.87	8.31
HLC ~ 1 + Eggs/site/day + Eggs/site/day:Site+Site+ Rain lag 3 + Rain lag 3^2	508.89	9.12
HLC ~ 1 + Eggs/site/day + Eggs/site/day:Site+Site+ Rain lag 2 + Rain lag 2^2	508.89	9.07
HLC ~ 1 + Eggs/site/day + Eggs/site/day:Site+Site+ Temperature lag 1 + Rain lag 3	508.90	9.20
HLC ~ 1 +Site+ Temperature + Rain lag 1	508.91	10.46
HLC ~ 1 + Eggs/site/day + Eggs/site/day:Site+Site+ Temperature lag 1 + Rain lag 2	508.93	9.19
HLC ~ 1 + Eggs/site/day + Temperature lag 2+ Temperature lag 2^2 + Rain lag 3 + Rain lag 3^2+Wind	508.93	8.65
HLC ~ 1 + Eggs/site/day + Eggs/site/day:Site+Site+ Temperature lag 2 + Rain lag 3+Wind	508.99	8.89
HLC ~ 1 + Eggs/site/day + Eggs/site/day:Site+Site+ Temperature lag 2 + Rain lag 2 +Wind	509.00	8.84
HLC ~ 1 + Eggs/site/day +Site+ Temperature lag 2 + Rain lag 3 + Rain lag 3^2+Wind	509.01	8.80
HLC ~ 1 + Eggs/site/day + Temperature lag 3+ Temperature lag 3^2 + Rain lag 3 + Rain lag 3^2+Wind	509.02	8.63
HLC ~ 1 + Eggs/site/day +Site+ Temperature + Rain lag 2 + Rain lag 2^2 +Wind	509.03	8.98
HLC ~ 1 + Eggs/site/day +Site+ Temperature lag 3 + Rain lag 3 + Rain lag 3^2	509.03	9.16
HLC ~ 1 + Eggs/site/day + Eggs/site/day:Site+Site+ Temperature lag 2+ Temperature lag 2^2 + Rain lag 1 +Wind	509.04	8.52
HLC ~ 1 + Eggs/site/day +Site+ Temperature lag 1 + Rain lag 2 + Rain lag 2^2 +Wind	509.05	8.90
HLC ~ 1 + Rain lag 1	509.06	10.58
HLC ~ 1 + Eggs/site/day +Site+ Temperature lag 3 + Rain lag 2 + Rain lag 2^2	509.06	9.09
HLC ~ 1 + Eggs/site/day + Eggs/site/day:Site+Site+ Rain lag 2 + Rain lag 2^2 +Wind	509.06	8.82
HLC ~ 1 + Eggs/site/day + Eggs/site/day:Site+Site+ Temperature lag 2+ Temperature lag 2^2 + Rain Index +Wind	509.09	8.26
HLC ~ 1 + Eggs/site/day + Temperature lag 3+ Temperature lag 3^2 + Rain lag 2 + Rain lag 2^2 +Wind	509.15	8.60
HLC ~ 1 + Eggs/site/day +Site+ Temperature lag 1 + Rain lag 2 + Rain lag 2^2	509.15	9.12
HLC ~ 1 + Eggs/site/day +Site+ Temperature lag 2 + Rain lag 2 + Rain lag 2^2	509.17	9.07

HLC ~ 1 + Eggs/site/day +Site+ Temperature lag 3+ Temperature lag 3^2 + Rain lag 1 + Rain lag 1^2	509.18	8.92
HLC ~ 1 + Eggs/site/day +Site+ Temperature lag 2 + Rain lag 3 + Rain lag 3^2	509.18	9.16
HLC ~ 1 + Eggs/site/day + Eggs/site/day:Site+Site+ Temperature lag 2+ Temperature lag 2^2 + Rain lag 2 +Wind	509.18	8.21
HLC ~ 1 + Eggs/site/day + Eggs/site/day:Site+Site+ Temperature lag 3+ Temperature lag 3^2 + Rain Index +Wind	509.21	8.29
HLC ~ 1 + Eggs/site/day +Site+ Temperature lag 2+ Temperature lag 2^2 + Rain lag 2 + Rain lag 2^2 +Wind	509.22	8.11
HLC ~ 1 + Temperature lag 1+ Temperature lag 1^2 +Wind	509.22	9.42
HLC ~ 1 + Eggs/site/day + Eggs/site/day:Site+Site+ Temperature lag 2+ Temperature lag 2^2 + Rain lag 1	509.25	8.87
HLC ~ 1 + Eggs/site/day + Temperature lag 3+ Temperature lag 3^2 + Rain lag 2 + Rain lag 2^2	509.26	8.94
HLC ~ 1 + Eggs/site/day +Site+ Temperature lag 3 + Rain lag 2 + Rain lag 2^2 +Wind	509.27	8.85
HLC ~ 1 +Site+Wind	509.27	9.98
HLC ~ 1 +Site+ Temperature + Temperature^2 +Wind	509.28	9.24
HLC ~ 1 + Eggs/site/day + Eggs/site/day:Site+Site+ Temperature + Rain lag 1 + Rain lag 1^2 +Wind	509.32	9.07
HLC ~ 1 + Temperature + Rain lag 1 +Wind	509.32	10.52
HLC ~ 1 + Eggs/site/day +Site+ Temperature lag 1 + Rain lag 3 + Rain lag 3^2	509.34	9.18
HLC ~ 1 + Eggs/site/day +Site+ Temperature lag 2+ Temperature lag 2^2 + Rain lag 2 + Rain lag 2^2	509.39	8.45
HLC ~ 1 + Eggs/site/day +Site+ Temperature lag 2 + Rain lag 2 + Rain lag 2^2 +Wind	509.40	8.85
HLC ~ 1 + Eggs/site/day + Eggs/site/day:Site+Site+ Temperature lag 2+ Temperature lag 2^2 + Rain lag 3+Wind	509.41	8.32
HLC ~ 1 + Eggs/site/day + Eggs/site/day:Site+Site+ Temperature lag 3+ Temperature lag 3^2 + Rain lag 2 +Wind	509.41	8.26
HLC ~ 1 + Eggs/site/day + Temperature lag 2+ Temperature lag 2^2 + Rain lag 3 + Rain lag 3^2	509.44	9.11
HLC ~ 1 + Eggs/site/day + Eggs/site/day:Site+Site+ Temperature lag 3+ Temperature lag 3^2 + Rain lag 1 +Wind	509.45	8.68
HLC ~ 1 + Eggs/site/day + Eggs/site/day:Site+Site+ Temperature lag 1+ Temperature lag 1^2 + Rain lag 1 + Rain lag 1^2	509.46	8.23
HLC ~ 1 + Eggs/site/day + Eggs/site/day:Site+Site+ Temperature lag 3+ Temperature lag 3^2 + Rain lag 3+Wind	509.52	8.31
HLC ~ 1 + Temperature lag 1 + Rain Index	509.53	10.33
HLC ~ 1 + Temperature lag 2	509.54	10.44
HLC ~ 1 + Eggs/site/day + Eggs/site/day:Site+Site+ Temperature lag 3+ Temperature lag 3^2 + Rain lag 1	509.54	9.07
HLC ~ 1 + Eggs/site/day + Eggs/site/day:Site+Site+ Temperature lag 1 + Rain lag 1 + Rain lag 1^2 +Wind	509.54	8.88
HLC ~ 1 + Eggs/site/day + Eggs/site/day:Site+Site+ Temperature lag 3 + Rain lag 1 + Rain lag 1^2	509.58	9.11
HLC ~ 1 + Rain lag 3	509.59	10.49
HLC ~ 1 + Temperature lag 3	509.59	10.45
HLC ~ 1 + Rain lag 2	509.59	10.46
HLC ~ 1 + Rain lag 1 +Wind	509.59	10.34
HLC ~ 1 + Temperature + Rain lag 1 + Rain lag 1^2	509.65	10.68
HLC ~ 1 + Temperature lag 1+ Temperature lag 1^2	509.66	9.80

HLC ~ 1 + Eggs/site/day + Temperature lag 3+ Temperature lag 3^2 + Rain lag 3 + Rain lag 3^2	509.67	9.16
HLC ~ 1 + Eggs/site/day + Eggs/site/day:Site+Site+ Temperature lag 2+ Temperature lag 2^2 + Rain lag 2	509.69	8.63
HLC ~ 1 +Site+ Temperature + Temperature^2 + Rain lag 1	509.71	10.00
HLC ~ 1 +Site+ Temperature +Wind	509.71	9.98
HLC ~ 1 + Eggs/site/day +Site+ Temperature lag 3+ Temperature lag 3^2 + Rain lag 2 + Rain lag 2^2 +Wind	509.72	8.25
HLC ~ 1 + Eggs/site/day +Site+ Temperature lag 2+ Temperature lag 2^2 + Rain lag 3 + Rain lag 3^2+Wind	509.73	8.31
HLC ~ 1 +Site+ Temperature + Temperature^2 + Rain Index	509.74	9.40
HLC ~ 1 + Eggs/site/day +Site+ Temperature lag 3+ Temperature lag 3^2 + Rain lag 3 + Rain lag 3^2+Wind	509.74	8.30
HLC ~ 1 + Eggs/site/day + Eggs/site/day:Site+Site+ Temperature lag 3 + Rain lag 1 + Rain lag 1^2 +Wind	509.75	8.83
HLC ~ 1 + Eggs/site/day + Eggs/site/day:Site+Site+ Temperature lag 1 + Rain lag 1 + Rain lag 1^2	509.78	9.15
HLC ~ 1 + Temperature + Temperature^2 + Rain lag 2 +Wind	509.80	9.13
HLC ~ 1 + Eggs/site/day + Eggs/site/day:Site+Site+ Temperature lag 2 + Rain lag 1 + Rain lag 1^2	509.83	9.10
HLC ~ 1 + Eggs/site/day + Eggs/site/day:Site+Site+ Temperature lag 2+ Temperature lag 2^2 + Rain lag 3	509.84	8.77
HLC ~ 1 + Temperature + Temperature^2 + Rain lag 1 +Wind	509.85	9.87
HLC ~ 1 + Temperature lag 2 +Wind	509.87	10.19
HLC ~ 1 +Site+ Temperature lag 1 +Wind	509.90	9.88
HLC ~ 1 + Rain Index +Wind	509.92	10.21
HLC ~ 1 +Site+ Temperature + Rain Index	509.93	10.08
HLC ~ 1 + Eggs/site/day + Eggs/site/day:Site+Site+ Temperature + Rain lag 3 + Rain lag 3^2	509.94	9.26
HLC ~ 1 + Eggs/site/day + Eggs/site/day:Site+Site+ Temperature lag 2 + Rain lag 1 + Rain lag 1^2 +Wind	509.95	8.84
HLC ~ 1 + Eggs/site/day + Eggs/site/day:Site+Site+ Temperature lag 3+ Temperature lag 3^2 + Rain lag 2	509.96	8.73
HLC ~ 1 +Site+ Temperature lag 1	509.97	10.14
HLC ~ 1 + Eggs/site/day + Eggs/site/day:Site+Site+ Temperature lag 1+ Temperature lag 1^2 + Rain lag 2 + Rain lag 2^2	509.99	7.97
HLC ~ 1 + Eggs/site/day + Eggs/site/day:Site+Site+ Temperature lag 1+ Temperature lag 1^2 + Rain lag 3 + Rain lag 3^2	510.00	8.02
HLC ~ 1 + Eggs/site/day +Site+ Temperature lag 3+ Temperature lag 3^2 + Rain lag 2 + Rain lag 2^2	510.00	8.63
HLC ~ 1 + Eggs/site/day + Eggs/site/day:Site+Site+ Temperature + Rain lag 2 + Rain lag 2^2	510.01	9.27
HLC ~ 1 + Temperature lag 3 +Wind	510.05	10.21
HLC ~ 1 + Temperature + Temperature^2 + Rain Index +Wind	510.07	9.42
HLC ~ 1 + Temperature + Temperature^2 + Rain lag 3 + Rain lag 3^2	510.09	9.19
HLC ~ 1 + Rain lag 2 +Wind	510.10	10.21
HLC ~ 1 + Eggs/site/day + Eggs/site/day:Site+Site+ Temperature lag 3+ Temperature lag 3^2 + Rain lag 3	510.10	8.86
HLC ~ 1 + Rain lag 3+Wind	510.10	10.23
HLC ~ 1 +Site+ Temperature + Temperature^2 + Rain lag 2	510.13	9.50

HLC ~ 1 + Temperature lag 1 + Rain lag 1 +Wind	510.17	10.29
HLC ~ 1 + Temperature lag 1 + Rain lag 1	510.17	10.54
HLC ~ 1 +Site+ Temperature + Rain lag 2	510.26	10.31
HLC ~ 1 +Site+ Temperature + Rain lag 3	510.26	10.21
HLC ~ 1 + Temperature + Rain lag 3 + Rain lag 3^2	510.27	10.27
HLC ~ 1 +Site+ Rain Index	510.31	10.18
HLC ~ 1 + Temperature lag 1 + Rain Index +Wind	510.36	10.13
HLC ~ 1 + Temperature + Temperature^2 + Rain lag 1 + Rain lag 1^2	510.40	10.06
HLC ~ 1 + Temperature + Rain Index +Wind	510.41	10.21
HLC ~ 1 + Eggs/site/day +Site+ Temperature lag 2+ Temperature lag 2^2 + Rain lag 3 + Rain lag 3^2	510.49	8.81
HLC ~ 1 + Temperature + Rain lag 2 +Wind	510.53	10.32
HLC ~ 1 + Temperature + Rain lag 3+Wind	510.53	10.24
HLC ~ 1 + Temperature + Temperature^2 + Rain lag 3 + Rain lag 3^2+Wind	510.57	8.60
HLC ~ 1 +Site+ Temperature lag 1+ Temperature lag 1^2 +Wind	510.57	9.21
HLC ~ 1 +Site+ Rain lag 1	510.60	10.37
HLC ~ 1 + Eggs/site/day +Site+ Temperature lag 3+ Temperature lag 3^2 + Rain lag 3 + Rain lag 3^2	510.65	8.91
HLC ~ 1 + Temperature lag 1 + Rain lag 3+Wind	510.66	10.05
HLC ~ 1 + Eggs/site/day + Eggs/site/day:Site+Site+ Temperature lag 2+ Temperature lag 2^2 + Rain lag 1 + Rain lag 1^2 +Wind	510.67	8.27
HLC ~ 1 + Temperature lag 1 + Rain lag 2 +Wind	510.74	10.16
HLC ~ 1 + Temperature lag 1 + Rain lag 3	510.74	10.36
HLC ~ 1 + Temperature lag 1 + Rain lag 2	510.76	10.41
HLC ~ 1 +Site+ Temperature + Temperature^2 + Rain lag 2 +Wind	510.77	8.95
HLC ~ 1 + Temperature lag 2+ Temperature lag 2^2	510.89	10.01
HLC ~ 1 + Temperature + Rain lag 2 + Rain lag 2^2	510.94	10.54
HLC ~ 1 + Temperature lag 2+ Temperature lag 2^2 +Wind	510.96	9.65
HLC ~ 1 + Temperature lag 1+ Temperature lag 1^2 + Rain lag 3+Wind	510.97	9.09
HLC ~ 1 + Temperature lag 2 + Rain Index	510.98	10.40
HLC ~ 1 + Eggs/site/day + Eggs/site/day:Site+Site+ Temperature + Rain lag 3 + Rain lag 3^2+Wind	510.99	8.90
HLC ~ 1 +Site+ Temperature + Rain lag 1 +Wind	511.00	10.32
HLC ~ 1 + Eggs/site/day + Eggs/site/day:Site+Site+ Temperature lag 2+ Temperature lag 2^2 + Rain lag 1 + Rain lag 1^2	511.03	8.66
HLC ~ 1 +Site+ Temperature lag 1+ Temperature lag 1^2	511.05	9.61
HLC ~ 1 +Site+ Temperature lag 2	511.05	10.22
HLC ~ 1 +Site+ Temperature lag 1 + Rain Index	511.09	10.09
HLC ~ 1 + Temperature lag 3 + Rain Index	511.09	10.41
HLC ~ 1 +Site+ Rain lag 2	511.10	10.26
HLC ~ 1 +Site+ Temperature lag 3	511.11	10.24
HLC ~ 1 + Eggs/site/day + Eggs/site/day:Site+Site+ Temperature lag 3 + Rain lag 3 + Rain lag 3^2	511.11	9.13
HLC ~ 1 +Site+ Rain lag 3	511.11	10.29
HLC ~ 1 + Eggs/site/day + Eggs/site/day:Site+Site+ Temperature lag 3 + Rain lag 3 + Rain lag 3^2+Wind	511.15	8.78

HLC ~ 1 + Eggs/site/day + Eggs/site/day:Site+Site+ Temperature lag 3+ Temperature lag 3^2 + Rain lag 1 + Rain lag 1^2 +Wind	511.16	8.41
HLC ~ 1 +Site+ Rain lag 1 +Wind	511.16	10.12
HLC ~ 1 + Eggs/site/day + Eggs/site/day:Site+Site+ Temperature lag 3 + Rain lag 2 + Rain lag 2^2	511.18	9.07
HLC ~ 1 + Temperature lag 1+ Temperature lag 1^2 + Rain Index	511.19	9.86
HLC ~ 1 + Eggs/site/day + Eggs/site/day:Site+Site+ Temperature lag 1 + Rain lag 3 + Rain lag 3^2+Wind	511.19	8.82
HLC ~ 1 + Temperature lag 3+ Temperature lag 3^2	511.22	10.08
HLC ~ 1 + Temperature + Temperature^2 + Rain lag 2 + Rain lag 2^2	511.23	9.68
HLC ~ 1 + Eggs/site/day + Eggs/site/day:Site+Site+ Temperature lag 2 + Rain lag 3 + Rain lag 3^2	511.24	9.14
HLC ~ 1 + Temperature lag 2 + Rain lag 1	511.26	10.59
HLC ~ 1 + Temperature lag 1+ Temperature lag 1^2 + Rain lag 2 +Wind	511.27	9.20
HLC ~ 1 + Eggs/site/day + Eggs/site/day:Site+Site+ Temperature lag 2 + Rain lag 2 + Rain lag 2^2	511.29	9.06
HLC ~ 1 + Eggs/site/day + Eggs/site/day:Site+Site+ Temperature lag 1 + Rain lag 2 + Rain lag 2^2	511.31	9.12
HLC ~ 1 + Rain lag 1 + Rain lag 1^2	511.32	10.56
HLC ~ 1 + Eggs/site/day + Eggs/site/day:Site+Site+ Temperature lag 2 + Rain lag 3 + Rain lag 3^2+Wind	511.33	8.79
HLC ~ 1 +Site+ Temperature + Temperature^2 + Rain lag 3 + Rain lag 3^2	511.34	8.98
HLC ~ 1 + Eggs/site/day + Eggs/site/day:Site+Site+ Temperature + Rain lag 2 + Rain lag 2^2 +Wind	511.34	8.98
HLC ~ 1 + Temperature lag 3+ Temperature lag 3^2 +Wind	511.35	9.68
HLC ~ 1 +Site+ Temperature + Rain lag 1 + Rain lag 1^2	511.36	10.49
HLC ~ 1 + Temperature lag 3 + Rain lag 1	511.36	10.60
HLC ~ 1 + Eggs/site/day + Eggs/site/day:Site+Site+ Temperature lag 1 + Rain lag 2 + Rain lag 2^2 +Wind	511.37	8.90
HLC ~ 1 + Rain lag 3 + Rain lag 3^2	511.39	10.34
HLC ~ 1 + Eggs/site/day + Eggs/site/day:Site+Site+ Temperature lag 3+ Temperature lag 3^2 + Rain lag 1 + Rain lag 1^2	511.41	8.83
HLC ~ 1 +Site+ Temperature lag 2 +Wind	511.41	9.96
HLC ~ 1 +Site+ Temperature + Temperature^2 + Rain lag 1 +Wind	511.41	9.70
HLC ~ 1 + Eggs/site/day + Eggs/site/day:Site+Site+ Temperature lag 1 + Rain lag 3 + Rain lag 3^2	511.43	9.16
HLC ~ 1 +Site+ Temperature + Temperature^2 + Rain lag 3 + Rain lag 3^2+Wind	511.45	8.34
HLC ~ 1 +Site+ Rain Index +Wind	511.46	9.99
HLC ~ 1 + Temperature lag 1+ Temperature lag 1^2 + Rain lag 1 +Wind	511.48	9.64
HLC ~ 1 +Site+ Temperature + Temperature^2 + Rain Index +Wind	511.49	9.19
HLC ~ 1 + Eggs/site/day + Eggs/site/day:Site+Site+ Temperature lag 2+ Temperature lag 2^2 + Rain lag 2 + Rain lag 2^2	511.51	8.41
HLC ~ 1 + Eggs/site/day + Eggs/site/day:Site+Site+ Temperature lag 2+ Temperature lag 2^2 + Rain lag 2 + Rain lag 2^2 +Wind	511.52	8.09
HLC ~ 1 + Eggs/site/day + Eggs/site/day:Site+Site+ Temperature lag 3 + Rain lag 2 + Rain lag 2^2 +Wind	511.55	8.83
HLC ~ 1 + Temperature lag 1+ Temperature lag 1^2 + Rain Index +Wind	511.57	9.46
HLC ~ 1 +Site+ Temperature lag 3 +Wind	511.59	9.99
HLC ~ 1 +Site+ Rain lag 2 +Wind	511.63	10.00

HLC ~ 1 +Site+ Rain lag 3+Wind	511.64	10.02
HLC ~ 1 + Temperature lag 2 + Rain lag 1 +Wind	511.65	10.36
HLC ~ 1 + Rain lag 3 + Rain lag 3^2+Wind	511.68	10.02
HLC ~ 1 + Eggs/site/day + Eggs/site/day:Site+Site+ Temperature lag 2 + Rain lag 2 + Rain lag 2^2 +Wind	511.69	8.84
HLC ~ 1 + Temperature lag 1+ Temperature lag 1^2 + Rain lag 1	511.77	10.03
HLC ~ 1 + Temperature lag 1+ Temperature lag 1^2 + Rain lag 3	511.77	9.67
HLC ~ 1 + Temperature + Rain lag 1 + Rain lag 1^2 +Wind	511.77	10.53
HLC ~ 1 +Site+ Temperature lag 1 + Rain lag 1	511.78	10.32
HLC ~ 1 + Rain lag 2 + Rain lag 2^2	511.78	10.47
HLC ~ 1 +Site+ Temperature lag 1 + Rain lag 1 +Wind	511.81	10.07
HLC ~ 1 + Temperature lag 1+ Temperature lag 1^2 + Rain lag 2	511.83	9.69
HLC ~ 1 + Temperature lag 2 + Rain lag 3	511.84	10.50
HLC ~ 1 + Temperature lag 2 + Rain lag 2	511.85	10.49
HLC ~ 1 + Temperature lag 3 + Rain lag 3	511.89	10.52
HLC ~ 1 + Temperature lag 3 + Rain lag 2	511.89	10.49
HLC ~ 1 +Site+ Temperature + Rain lag 3 + Rain lag 3^2	511.90	10.07
HLC ~ 1 + Rain lag 1 + Rain lag 1^2 +Wind	511.92	10.32
HLC ~ 1 + Temperature lag 3 + Rain lag 1 +Wind	511.94	10.35
HLC ~ 1 +Site+ Temperature lag 1 + Rain Index +Wind	511.97	9.89
HLC ~ 1 + Eggs/site/day + Eggs/site/day:Site+Site+ Temperature lag 2+ Temperature lag 2^2 + Rain lag 3 + Rain lag 3^2+Wind	511.97	8.27
HLC ~ 1 + Rain lag 2 + Rain lag 2^2 +Wind	511.99	10.20
HLC ~ 1 +Site+ Temperature + Temperature^2 + Rain lag 1 + Rain lag 1^2	512.01	9.89
HLC ~ 1 + Eggs/site/day + Eggs/site/day:Site+Site+ Temperature lag 3+ Temperature lag 3^2 + Rain lag 3 + Rain lag 3^2+Wind	512.02	8.25
HLC ~ 1 + Eggs/site/day + Eggs/site/day:Site+Site+ Temperature lag 3+ Temperature lag 3^2 + Rain lag 2 + Rain lag 2^2 +Wind	512.03	8.22
HLC ~ 1 + Temperature lag 2 + Rain Index +Wind	512.03	10.20
HLC ~ 1 +Site+ Temperature + Rain Index +Wind	512.03	9.97
HLC ~ 1 + Temperature + Temperature^2 + Rain lag 1 + Rain lag 1^2 +Wind	512.04	9.66
HLC ~ 1 + Temperature + Rain lag 3 + Rain lag 3^2+Wind	512.05	10.06
HLC ~ 1 +Site+ Temperature lag 1+ Temperature lag 1^2 + Rain lag 3+Wind	512.13	8.91
HLC ~ 1 + Eggs/site/day + Eggs/site/day:Site+Site+ Temperature lag 3+ Temperature lag 3^2 + Rain lag 2 + Rain lag 2^2	512.14	8.59
HLC ~ 1 +Site+ Temperature + Rain lag 3+Wind	512.14	10.04
HLC ~ 1 +Site+ Temperature + Rain lag 2 +Wind	512.16	10.13
HLC ~ 1 + Temperature lag 3 + Rain Index +Wind	512.24	10.22
HLC ~ 1 +Site+ Temperature lag 1 + Rain lag 3+Wind	512.24	9.83
HLC ~ 1 + Temperature lag 2 + Rain lag 3+Wind	512.25	10.22
HLC ~ 1 + Temperature lag 2 + Rain lag 2 +Wind	512.25	10.25
HLC ~ 1 + Temperature lag 1+ Temperature lag 1^2 + Rain lag 3 + Rain lag 3^2+Wind	512.29	8.73
HLC ~ 1 + Temperature + Temperature^2 + Rain lag 2 + Rain lag 2^2 +Wind	512.29	9.17
HLC ~ 1 +Site+ Temperature lag 1 + Rain lag 3	512.31	10.15
HLC ~ 1 +Site+ Temperature lag 1 + Rain lag 2	512.34	10.21
HLC ~ 1 +Site+ Temperature lag 1 + Rain lag 2 +Wind	512.34	9.95
HLC ~ 1 +Site+ Temperature lag 2+ Temperature lag 2^2	512.36	9.82

HLC ~ 1 +Site+ Temperature lag 2+ Temperature lag 2^2 +Wind	512.40	9.45
HLC ~ 1 + Temperature lag 3 + Rain lag 2 +Wind	512.43	10.25
HLC ~ 1 + Temperature lag 3 + Rain lag 3+Wind	512.43	10.25
HLC ~ 1 +Site+ Temperature lag 1+ Temperature lag 1^2 + Rain lag 2 +Wind	512.46	9.04
HLC ~ 1 + Eggs/site/day + Eggs/site/day:Site+Site+ Temperature lag 2+ Temperature lag 2^2 + Rain lag 3 + Rain lag 3^2	512.47	8.75
HLC ~ 1 + Temperature lag 1 + Rain lag 1 + Rain lag 1^2	512.48	10.50
HLC ~ 1 + Temperature lag 1 + Rain lag 1 + Rain lag 1^2 +Wind	512.53	10.25
HLC ~ 1 +Site+ Temperature lag 2 + Rain Index	512.55	10.17
HLC ~ 1 + Temperature lag 2+ Temperature lag 2^2 + Rain Index	512.59	10.00
HLC ~ 1 +Site+ Temperature + Rain lag 2 + Rain lag 2^2	512.62	10.37
HLC ~ 1 +Site+ Temperature + Temperature^2 + Rain lag 2 + Rain lag 2^2	512.64	9.54
HLC ~ 1 +Site+ Temperature lag 1+ Temperature lag 1^2 + Rain Index	512.66	9.68
HLC ~ 1 +Site+ Temperature lag 3 + Rain Index	512.66	10.19
HLC ~ 1 +Site+ Temperature lag 3+ Temperature lag 3^2	512.67	9.94
HLC ~ 1 + Eggs/site/day + Eggs/site/day:Site+Site+ Temperature lag 3+ Temperature lag 3^2 + Rain lag 3 + Rain lag 3^2	512.68	8.83
HLC ~ 1 + Temperature + Rain lag 2 + Rain lag 2^2 +Wind	512.71	10.34
HLC ~ 1 + Temperature lag 3+ Temperature lag 3^2 + Rain Index	512.74	9.99
HLC ~ 1 + Temperature lag 1 + Rain lag 3 + Rain lag 3^2+Wind	512.76	9.94
HLC ~ 1 +Site+ Temperature lag 3+ Temperature lag 3^2 +Wind	512.76	9.50
HLC ~ 1 +Site+ Temperature lag 2 + Rain lag 1	512.87	10.38
HLC ~ 1 + Temperature lag 1 + Rain lag 3 + Rain lag 3^2	512.91	10.28
HLC ~ 1 +Site+ Rain lag 1 + Rain lag 1^2	512.92	10.35
HLC ~ 1 + Temperature lag 1 + Rain lag 2 + Rain lag 2^2 +Wind	512.93	10.18
HLC ~ 1 +Site+ Rain lag 3 + Rain lag 3^2	512.94	10.14
HLC ~ 1 +Site+ Temperature lag 1+ Temperature lag 1^2 + Rain lag 1 +Wind	512.96	9.47
HLC ~ 1 + Temperature lag 2+ Temperature lag 2^2 + Rain lag 1	512.97	10.21
HLC ~ 1 +Site+ Temperature lag 3 + Rain lag 1	512.97	10.40
HLC ~ 1 +Site+ Temperature lag 1+ Temperature lag 1^2 + Rain Index +Wind	513.01	9.26
HLC ~ 1 + Temperature lag 1 + Rain lag 2 + Rain lag 2^2	513.11	10.42
HLC ~ 1 +Site+ Temperature lag 1+ Temperature lag 1^2 + Rain lag 3	513.13	9.51
HLC ~ 1 + Temperature lag 2+ Temperature lag 2^2 + Rain lag 1 +Wind	513.16	9.86
HLC ~ 1 +Site+ Temperature lag 1+ Temperature lag 1^2 + Rain lag 2	513.18	9.56
HLC ~ 1 + Temperature lag 2+ Temperature lag 2^2 + Rain lag 2	513.20	10.00
HLC ~ 1 +Site+ Temperature lag 1+ Temperature lag 1^2 + Rain lag 3 + Rain lag 3^2+Wind	513.22	8.51
HLC ~ 1 +Site+ Rain lag 3 + Rain lag 3^2+Wind	513.23	9.80
HLC ~ 1 + Temperature lag 2+ Temperature lag 2^2 + Rain lag 3	513.24	10.04
HLC ~ 1 + Temperature lag 3+ Temperature lag 3^2 + Rain lag 3+Wind	513.27	9.47
HLC ~ 1 + Temperature lag 2+ Temperature lag 2^2 + Rain lag 3+Wind	513.27	9.60
HLC ~ 1 + Temperature lag 2+ Temperature lag 2^2 + Rain lag 2 +Wind	513.28	9.60
HLC ~ 1 +Site+ Temperature lag 1+ Temperature lag 1^2 + Rain lag 1	513.28	9.88
HLC ~ 1 +Site+ Temperature lag 2 + Rain lag 1 +Wind	513.29	10.14
HLC ~ 1 + Temperature lag 2+ Temperature lag 2^2 + Rain Index +Wind	513.32	9.67
HLC ~ 1 +Site+ Temperature + Temperature^2 + Rain lag 2 + Rain lag 2^2 +Wind	513.35	9.02
HLC ~ 1 + Temperature lag 3+ Temperature lag 3^2 + Rain lag 1	513.37	10.37

HLC ~ 1 +Site+ Rain lag 2 + Rain lag 2^2	513.37	10.27
HLC ~ 1 + Temperature lag 3+ Temperature lag 3^2 + Rain lag 2 +Wind	513.42	9.54
HLC ~ 1 + Temperature lag 3+ Temperature lag 3^2 + Rain lag 3	513.42	10.08
HLC ~ 1 + Temperature lag 3+ Temperature lag 3^2 + Rain lag 2	513.43	10.05
HLC ~ 1 +Site+ Temperature lag 2 + Rain lag 2	513.43	10.29
HLC ~ 1 +Site+ Temperature lag 2 + Rain lag 3	513.43	10.30
HLC ~ 1 +Site+ Temperature lag 3 + Rain lag 2	513.48	10.31
HLC ~ 1 +Site+ Temperature lag 3 + Rain lag 3	513.48	10.33
HLC ~ 1 + Temperature lag 1+ Temperature lag 1^2 + Rain lag 2 + Rain lag 2^2 +Wind	513.50	9.19
HLC ~ 1 +Site+ Temperature + Rain lag 1 + Rain lag 1^2 +Wind	513.53	10.34
HLC ~ 1 +Site+ Rain lag 1 + Rain lag 1^2 +Wind	513.56	10.10
HLC ~ 1 +Site+ Temperature lag 3 + Rain lag 1 +Wind	513.57	10.14
HLC ~ 1 +Site+ Temperature + Temperature^2 + Rain lag 1 + Rain lag 1^2 +Wind	513.57	9.49
HLC ~ 1 + Temperature lag 2 + Rain lag 1 + Rain lag 1^2	513.59	10.57
HLC ~ 1 + Temperature lag 1+ Temperature lag 1^2 + Rain lag 3 + Rain lag 3^2	513.59	9.44
HLC ~ 1 +Site+ Rain lag 2 + Rain lag 2^2 +Wind	513.61	10.00
HLC ~ 1 + Temperature lag 3+ Temperature lag 3^2 + Rain Index +Wind	513.62	9.67
HLC ~ 1 +Site+ Temperature lag 2 + Rain Index +Wind	513.63	9.97
HLC ~ 1 + Temperature lag 3+ Temperature lag 3^2 + Rain lag 1 +Wind	513.68	9.93
HLC ~ 1 + Temperature lag 3 + Rain lag 1 + Rain lag 1^2	513.70	10.58
HLC ~ 1 +Site+ Temperature + Rain lag 3 + Rain lag 3^2+Wind	513.70	9.85
HLC ~ 1 + Temperature lag 2 + Rain lag 3 + Rain lag 3^2	513.74	10.36
HLC ~ 1 + Temperature lag 1+ Temperature lag 1^2 + Rain lag 1 + Rain lag 1^2 +Wind	513.76	9.51
HLC ~ 1 + Temperature lag 3 + Rain lag 3 + Rain lag 3^2	513.77	10.38
HLC ~ 1 +Site+ Temperature lag 3 + Rain Index +Wind	513.85	9.99
HLC ~ 1 +Site+ Temperature lag 2 + Rain lag 3+Wind	513.85	10.01
HLC ~ 1 +Site+ Temperature lag 2 + Rain lag 2 +Wind	513.86	10.05
HLC ~ 1 + Temperature lag 2 + Rain lag 3 + Rain lag 3^2+Wind	513.95	10.03
HLC ~ 1 + Temperature lag 1+ Temperature lag 1^2 + Rain lag 1 + Rain lag 1^2	514.03	9.93
HLC ~ 1 +Site+ Temperature lag 3 + Rain lag 2 +Wind	514.03	10.05
HLC ~ 1 + Temperature lag 2 + Rain lag 1 + Rain lag 1^2 +Wind	514.04	10.33
HLC ~ 1 +Site+ Temperature lag 3 + Rain lag 3+Wind	514.04	10.05
HLC ~ 1 + Temperature lag 3 + Rain lag 3 + Rain lag 3^2+Wind	514.11	10.06
HLC ~ 1 + Temperature lag 2 + Rain lag 2 + Rain lag 2^2	514.12	10.50
HLC ~ 1 +Site+ Temperature lag 2+ Temperature lag 2^2 + Rain Index	514.12	9.81
HLC ~ 1 +Site+ Temperature lag 1 + Rain lag 1 + Rain lag 1^2	514.15	10.28
HLC ~ 1 + Temperature lag 3 + Rain lag 2 + Rain lag 2^2	514.16	10.50
HLC ~ 1 +Site+ Temperature lag 3+ Temperature lag 3^2 + Rain Index	514.22	9.84
HLC ~ 1 + Temperature lag 2 + Rain lag 2 + Rain lag 2^2 +Wind	514.23	10.26
HLC ~ 1 +Site+ Temperature lag 1 + Rain lag 1 + Rain lag 1^2 +Wind	514.23	10.03
HLC ~ 1 + Temperature lag 1+ Temperature lag 1^2 + Rain lag 2 + Rain lag 2^2	514.26	9.70
HLC ~ 1 + Temperature lag 3 + Rain lag 1 + Rain lag 1^2 +Wind	514.33	10.33
HLC ~ 1 +Site+ Temperature lag 1 + Rain lag 3 + Rain lag 3^2+Wind	514.37	9.72
HLC ~ 1 + Temperature lag 3 + Rain lag 2 + Rain lag 2^2 +Wind	514.41	10.25
HLC ~ 1 +Site+ Temperature + Rain lag 2 + Rain lag 2^2 +Wind	514.43	10.17
HLC ~ 1 +Site+ Temperature lag 3+ Temperature lag 3^2 + Rain lag 3+Wind	514.52	9.38

HLC ~ 1 +Site+ Temperature lag 1 + Rain lag 3 + Rain lag 3^2	514.53	10.07
HLC ~ 1 +Site+ Temperature lag 2+ Temperature lag 2^2 + Rain lag 1	514.55	10.04
HLC ~ 1 +Site+ Temperature lag 1 + Rain lag 2 + Rain lag 2^2 +Wind	514.61	9.99
HLC ~ 1 +Site+ Temperature lag 3+ Temperature lag 3^2 + Rain lag 2 +Wind	514.66	9.45
HLC ~ 1 +Site+ Temperature lag 2+ Temperature lag 2^2 + Rain lag 2	514.70	9.84
HLC ~ 1 +Site+ Temperature lag 2+ Temperature lag 2^2 + Rain lag 2 +Wind	514.71	9.45
HLC ~ 1 +Site+ Temperature lag 2+ Temperature lag 2^2 + Rain lag 3+Wind	514.71	9.44
HLC ~ 1 +Site+ Temperature lag 2+ Temperature lag 2^2 + Rain lag 1 +Wind	514.72	9.68
HLC ~ 1 +Site+ Temperature lag 2+ Temperature lag 2^2 + Rain lag 3	514.76	9.88
HLC ~ 1 +Site+ Temperature lag 1+ Temperature lag 1^2 + Rain lag 2 + Rain lag 2^2 +Wind	514.77	9.06
HLC ~ 1 +Site+ Temperature lag 1 + Rain lag 2 + Rain lag 2^2	514.77	10.23
HLC ~ 1 +Site+ Temperature lag 3+ Temperature lag 3^2 + Rain lag 2	514.83	10.00
HLC ~ 1 +Site+ Temperature lag 2+ Temperature lag 2^2 + Rain Index +Wind	514.84	9.47
HLC ~ 1 +Site+ Temperature lag 3+ Temperature lag 3^2 + Rain lag 3	514.84	10.06
HLC ~ 1 +Site+ Temperature lag 1+ Temperature lag 1^2 + Rain lag 3 + Rain lag 3^2	514.90	9.27
HLC ~ 1 +Site+ Temperature lag 3+ Temperature lag 3^2 + Rain lag 1	514.93	10.28
HLC ~ 1 +Site+ Temperature lag 3+ Temperature lag 3^2 + Rain Index +Wind	515.09	9.49
HLC ~ 1 + Temperature lag 3+ Temperature lag 3^2 + Rain lag 3 + Rain lag 3^2+Wind	515.15	9.36
HLC ~ 1 +Site+ Temperature lag 3+ Temperature lag 3^2 + Rain lag 1 +Wind	515.21	9.80
HLC ~ 1 + Temperature lag 2+ Temperature lag 2^2 + Rain lag 3 + Rain lag 3^2+Wind	515.22	9.50
HLC ~ 1 + Temperature lag 2+ Temperature lag 2^2 + Rain lag 1 + Rain lag 1^2	515.24	10.12
HLC ~ 1 +Site+ Temperature lag 2 + Rain lag 1 + Rain lag 1^2	515.26	10.35
HLC ~ 1 +Site+ Temperature lag 1+ Temperature lag 1^2 + Rain lag 1 + Rain lag 1^2 +Wind	515.27	9.34
HLC ~ 1 + Temperature lag 2+ Temperature lag 2^2 + Rain lag 3 + Rain lag 3^2	515.35	9.97
HLC ~ 1 +Site+ Temperature lag 2 + Rain lag 3 + Rain lag 3^2	515.36	10.16
HLC ~ 1 +Site+ Temperature lag 3 + Rain lag 1 + Rain lag 1^2	515.37	10.37
HLC ~ 1 +Site+ Temperature lag 3 + Rain lag 3 + Rain lag 3^2	515.39	10.19
HLC ~ 1 + Temperature lag 2+ Temperature lag 2^2 + Rain lag 1 + Rain lag 1^2 +Wind	515.44	9.74
HLC ~ 1 + Temperature lag 3+ Temperature lag 3^2 + Rain lag 3 + Rain lag 3^2	515.47	10.01
HLC ~ 1 +Site+ Temperature lag 2 + Rain lag 3 + Rain lag 3^2+Wind	515.57	9.82
HLC ~ 1 +Site+ Temperature lag 1+ Temperature lag 1^2 + Rain lag 1 + Rain lag 1^2	515.58	9.77
HLC ~ 1 + Temperature lag 3+ Temperature lag 3^2 + Rain lag 1 + Rain lag 1^2	515.64	10.25
HLC ~ 1 + Temperature lag 2+ Temperature lag 2^2 + Rain lag 2 + Rain lag 2^2	515.65	10.02
HLC ~ 1 +Site+ Temperature lag 1+ Temperature lag 1^2 + Rain lag 2 + Rain lag 2^2	515.69	9.57
HLC ~ 1 + Temperature lag 2+ Temperature lag 2^2 + Rain lag 2 + Rain lag 2^2 +Wind	515.70	9.71
HLC ~ 1 +Site+ Temperature lag 3 + Rain lag 3 + Rain lag 3^2+Wind	515.74	9.86
HLC ~ 1 +Site+ Temperature lag 2 + Rain lag 1 + Rain lag 1^2 +Wind	515.74	10.11
HLC ~ 1 + Temperature lag 3+ Temperature lag 3^2 + Rain lag 2 + Rain lag 2^2 +Wind	515.76	9.66
HLC ~ 1 +Site+ Temperature lag 2 + Rain lag 2 + Rain lag 2^2	515.78	10.31
HLC ~ 1 +Site+ Temperature lag 3 + Rain lag 2 + Rain lag 2^2	515.82	10.32
HLC ~ 1 + Temperature lag 3+ Temperature lag 3^2 + Rain lag 2 + Rain lag 2^2	515.86	10.12
HLC ~ 1 + Temperature lag 3+ Temperature lag 3^2 + Rain lag 1 + Rain lag 1^2 +Wind	515.91	9.77
HLC ~ 1 +Site+ Temperature lag 2 + Rain lag 2 + Rain lag 2^2 +Wind	515.93	10.08
HLC ~ 1 +Site+ Temperature lag 3 + Rain lag 1 + Rain lag 1^2 +Wind	516.04	10.12
HLC ~ 1 +Site+ Temperature lag 3 + Rain lag 2 + Rain lag 2^2 +Wind	516.10	10.07
HLC ~ 1 +Site+ Temperature lag 3+ Temperature lag 3^2 + Rain lag 3 + Rain lag 3^2+Wind	516.43	9.24

HLC ~ 1 +Site+ Temperature lag 2+ Temperature lag 2^2 + Rain lag 3 + Rain lag 3^2+Wind	516.69	9.32
HLC ~ 1 +Site+ Temperature lag 2+ Temperature lag 2^2 + Rain lag 1 + Rain lag 1^2	516.87	9.95
HLC ~ 1 +Site+ Temperature lag 2+ Temperature lag 2^2 + Rain lag 3 + Rain lag 3^2	516.92	9.81
HLC ~ 1 +Site+ Temperature lag 3+ Temperature lag 3^2 + Rain lag 3 + Rain lag 3^2	516.94	9.97
HLC ~ 1 +Site+ Temperature lag 2+ Temperature lag 2^2 + Rain lag 1 + Rain lag 1^2 +Wind	517.03	9.56
HLC ~ 1 +Site+ Temperature lag 3+ Temperature lag 3^2 + Rain lag 2 + Rain lag 2^2 +Wind	517.14	9.60
HLC ~ 1 +Site+ Temperature lag 2+ Temperature lag 2^2 + Rain lag 2 + Rain lag 2^2	517.23	9.89
HLC ~ 1 +Site+ Temperature lag 3+ Temperature lag 3^2 + Rain lag 1 + Rain lag 1^2	517.24	10.18
HLC ~ 1 +Site+ Temperature lag 2+ Temperature lag 2^2 + Rain lag 2 + Rain lag 2^2 +Wind	517.24	9.59
HLC ~ 1 +Site+ Temperature lag 3+ Temperature lag 3^2 + Rain lag 2 + Rain lag 2^2	517.35	10.08
HLC ~ 1 +Site+ Temperature lag 3+ Temperature lag 3^2 + Rain lag 1 + Rain lag 1^2 +Wind	517.44	9.65
