# Supplementary figures and images for: From eggs to bites: do ovitrap data provide reliable estimates of Aedes albopictus biting females?
Source: PeerJ. 2017 Mar 16;5:e2998. doi: 10.7717/peerj.2998 (PMC5357344; doi:10.7717/peerj.2998)

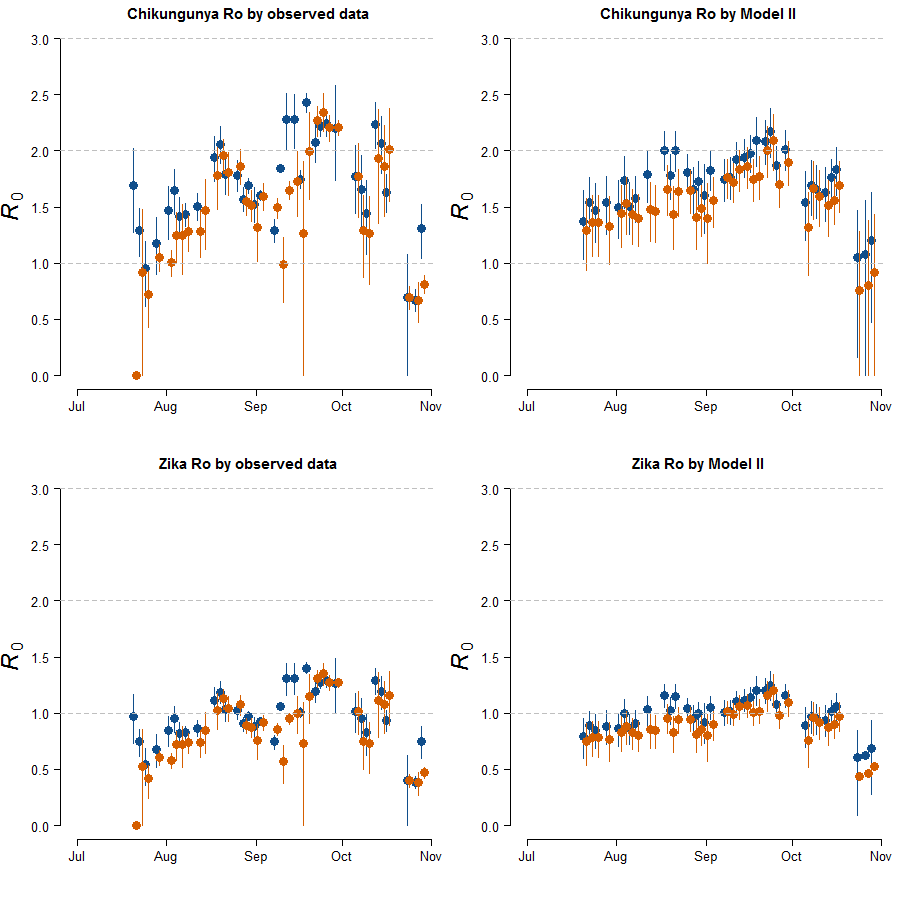

Supplement: Figure S1 — Estimated R0 based either on observed HLC data (A, B), or on the mean number eggs/site/day and its estimated relationship with biting Ae. albopictus females by Model-II (C, D). x-axis: months, y-axis: R0. Dots, mean values; solid lines, confidence intervals; blue, Site-1; red, site-2. [file peerj-05-2998-s010.png]

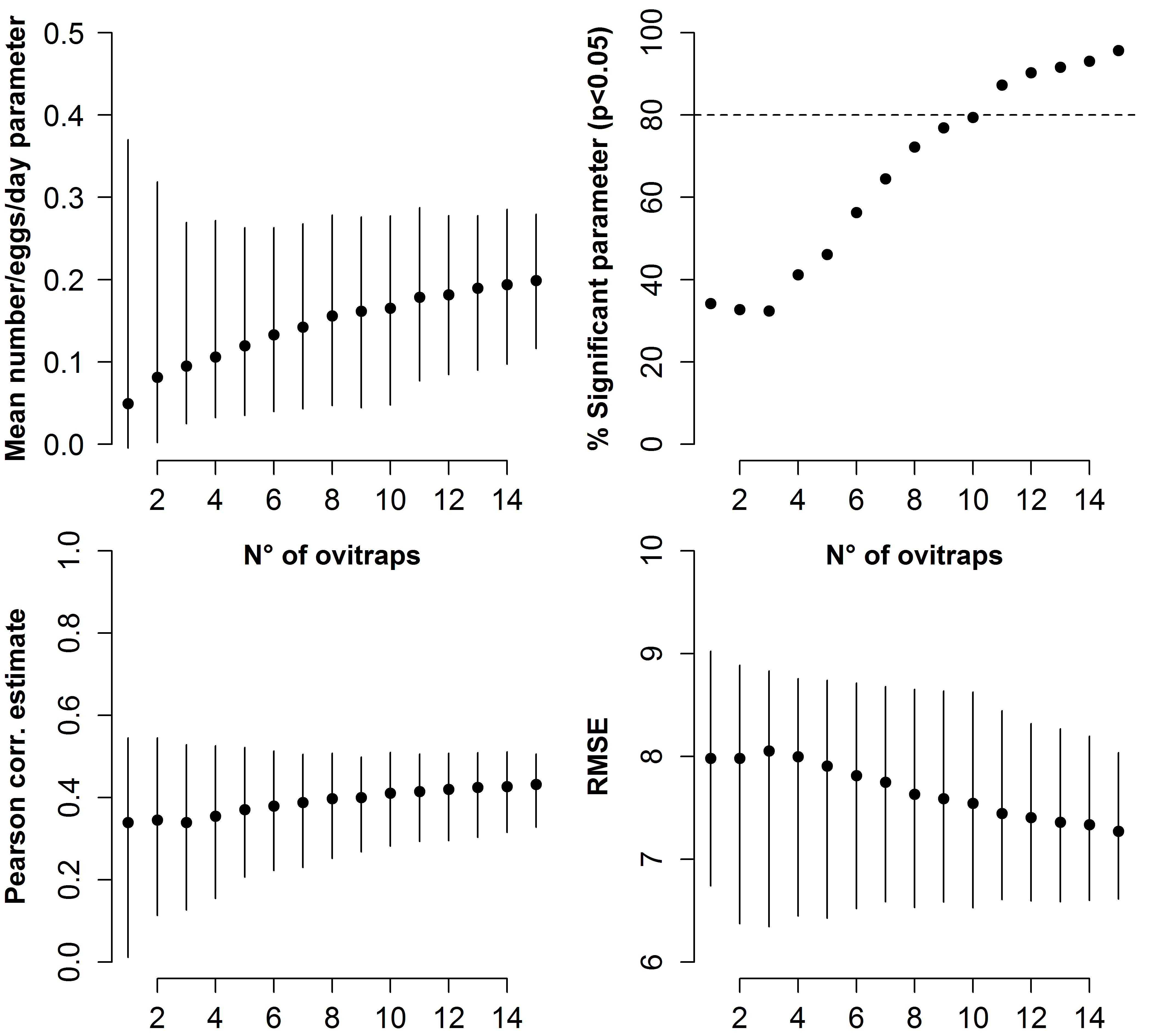

Supplement: Figure S2 — On the x axis the number of ovitraps. Dots represent mean values, vertical lines represent 95% confidence intervals based on 1,000 simulation. Upper left panel) estimated values of the mean number/eggs/days parameter in Model II. Upper right panel) Percentage of statistically significant ( p values < 0.05) mean number/eggs/days parameter. Lower left panel) estimated pearson correlation between between observed mean number/eggs/day and adult biting females. Lower right panel) Residual mean squared error (RMSE) of simulated Model II. [file peerj-05-2998-s011.png]
